# Presence of recombination hotspots throughout *SLC6A3*

**DOI:** 10.1371/journal.pone.0218129

**Published:** 2019-06-11

**Authors:** Juan Zhao, Yanhong Zhou, Nian Xiong, Hong Qing, Tao Wang, Zhicheng Lin

**Affiliations:** 1 School of Life Science, Beijing Institute of Technology, Beijing, China; 2 Laboratory of Psychiatric Neurogenomics, Basic Neuroscience Division, McLean Hospital, Belmont, MA, United States of America; 3 The Key Laboratory of Carcinogenesis of The Chinese Ministry of Health, Xiangya Hospital, Central South University, Changsha, Hunan, P.R. China; 4 Department of Neurology, Union Hospital, Tongji Medical College, Huazhong University of Science and Technology, Wuhan, Hubei, China; Sri Ramachandra Institute of Higher Education and Research, Chennai, India, INDIA

## Abstract

The human dopamine transporter gene *SLC6A3* is involved in substance use disorders (SUDs) among many other common neuropsychiatric illnesses but allelic association results including those with its classic genetic markers 3’VNTR or Int8VNTR remain mixed and unexplainable. To better understand the genetics for reproducible association signals, we report the presence of recombination hotspots based on sequencing of the entire 5’ promoter regions in two small SUDs cohorts, 30 African Americans (AAs) and 30 European Americans (EAs). Recombination rate was the highest near the transcription start site (TSS) in both cohorts. In addition, each cohort carried 57 different promoter haplotypes out of 60 and no haplotypes were shared between the two ethnicities. A quarter of the haplotypes evolved in an ethnicity-specific manner. Finally, analysis of five hundred subjects of European ancestry, from the 1000 Genome Project, confirmed the promoter recombination hotspots and also revealed several additional ones in non-coding regions only. These findings provide an explanation for the mixed results as well as guidance for selection of effective markers to be used in next generation association validation (NGAV), facilitating the delineation of pathogenic variation in this critical neuropsychiatric gene.

## Introduction

By sequestering dopamine (DA) into presynaptic neurons, the dopamine transporter (DAT) regulates spatio-temporal components of DA transmission. As a critical regulator of DA transmission, DAT contributes to voluntary movement, reward and mnemonic functions of the brain and modulates the efficacy of therapeutic drugs targeted to this plasma membrane protein. DAT expression is highly circumscribed in discrete regions throughout the brain and the expression of the human DAT gene (*SLC6A3*) varies among subjects[1–5]. Thus DNA sequence variations in the regulatory regions specially the promoter of *SLC6A3* may contribute to altered expressional patterns in the brain, dopamine-related individuality as well as diseases[6, 7]. The essential roles of DAT in brain function have mandated extensive studies of *SLC6A3* associations with behaviors and diseases.

During the last twenty five years *SLC6A3* has been extensively studied for genetic associations but the association studies with different markers located in *SLC6A3*’s 3’ regions obtained mixed results. On chromosome 5 (chr5), *SLC6A3* spans 70 kilobase (kb) from the 5’ promoter to 3’untranslated region (3’UTR, located in Exon 15). Sporadic genetic markers in several regulatory regions throughout *SLC6A3*, including the 5’ promoter, Intron 8 and 3’UTR, have been used in hundreds of association studies on more than eight different diseases and a number of human behaviors. Vast majority of these studies used a classical variable number tandem repeat located in the 3’UTR (3’VNTR/rs28363170) and another more recently used VNTR in Intron 8 (Int8VNTR/rs3836790)[8, 9]. As a result, findings from these association studies, especially those with 3’VNTR, in various populations were largely inconclusive or showed small effect sizes, such as studies on schizophrenia and bipolar disorder among others[10–15]. In particular, *SLC6A3* has been well implicated in the etiology and treatment of attention deficit hyperactivity disorder (ADHD)[16–22] but human genetic association studies with the 3’VNTR could not obtain consistent positive signals[23–25]. Another example was pharmacogenetics of Parkinson’s disease (PD): 3’VNTR or In8VNTR was associated with differential response to pharmacotherapy of PD[26–30] but not based on other studies [31, 32]. Moreover, *SLC6A3* genotype was found to modulate the risk of pesticide exposure for PD by some studies [33]but not by others[34]. The lack of evidence-based selection of markers resulted in the mixture, unfortunately causing little motivation to add more association studies with any markers in the *SLC6A3* genetic field. Importantly, the unreliable human genetic findings are inconsistent with ample positively-related evidence for *SLC6A3* activity *versus* phenotypes from other approaches such as pharmacology, imaging and animal genetics[20, 35–39].

In contrast to 3’VNTR, its promoter markers were more consistently associated with ADHD in various populations [40–47]. Consistently, we and others have shown that the 5’ promoter regions display varying regulatory activity and also in a haplotype-dependent manner *in vitro*[48–51]. Findings from rodent genetic studies have demonstrated the causality of reduced DAT activity on various phenotypes[37, 52–56]. These findings suggest that polymorphism- or haplotype-dependent *SLC6A3* promoter activity may confer risk for related diseases and that genetic association studies should have resulted in consistent positive findings.

One explanation for the current elusiveness of the association findings with the 3’VNTR was that this marker is far away from upstream regulatory regions, including Int8VNTR and the 50 kb-away 5’ promoter, and unable to capture the related information due to high recombination rates or weak linkage disequilibrium (LD). Other explanation is that different populations carry different frequencies of the same markers or even different disease loci. In either case, these already used genetic markers were unlikely the underlying disease loci. *In vitro* studies have shown polymorphisms in the 5’ core promoter, Int8VNTR and 3’VNTR all regulated promoter activity[49, 57–61]. This information suggests that it be critical for association studies to use genetic markers in all distinct regulatory regions, in order to capture variable *SLC6A3* expression as a whole and identify the underlying haplotypes and signaling pathways[51].

To clarify these possibilities, it is necessary to deeply sequence the regulatory regions for a better understanding of the *SLC6A3* genetic structure including the 3’ VNTRs, given the implications of *SLC6A3* in a spectrum of diseases and other behavioral characteristics. This task requires systemic discovery of polymorphisms and haplotypes in the regulatory regions, through targeted deep-sequencing that is helpful in discovery of novel functional loci or mutations in different fields[62–69]. In other words, the presence of multiple *SLC6A3* regulatory regions mandates mechanistic studies of the *SLC6A3* genetics, to help delineating functionally distinct *SLC6A3* haplotypes.

In this targeted deep-sequencing study, we uncover unique and common polymorphisms and haplotypes, and recombination hotspots for two major U.S. populations, African Americans (AAs) and European Americans (EAs), for finding generalization. The findings may help explain the mixed association findings[25, 70, 71] and instruct our future strategy for identifying disease loci in *SLC6A3*[29, 72, 73].

## Materials and methods

### Subjects

Sixty unrelated subjects were selected from the Collaborative Studies on Genetics of Alcoholism (COGA) pedigrees[74] and their de-identified genomic DNAs were provided by COGA through the Coriell Institute (NJ, U.S.A.) with the approval of National Institute on Alcohol Abuse and Alcoholism (NIAAA). Subjects gave their informed consent to the COGA study. From each of the COGA pedigrees, we selected the grandparents and their offspring’s spouses that came from outside the pedigree as unrelated subjects and the unrelatedness was verified by genomic control[75]. They included two cohorts: 30 AAs and 30 EAs. Each cohort consisted of 15 controls and 15 patients with substance use disorders (SUDs) (see Table 1). The 30 control subjects were all unaffected based upon the Diagnostic and Statistical Manual of Mental Disorders III Revision (DSM-IIIR), Feighner Criteria and International Classification of Diseases, Tenth Revision (ICD-10). All of the 30 affected subjects met at least two of the DSM-IIIR, Feighner Criteria and ICD-10 criteria for alcohol dependence. This study was approved by McLean Hospital Institutional Review Board.

**Table 1:** Demographic information on 60 COGA subjects used.

	AA (30)			EA (30)		
	Control (15)	Case (15)	Total	Control (15)	Case (15)	Total
Male	7	7	14	8	8	16
Female	8	8	16	7	7	14
Average age	40.4 ± 4.3	44.3 ±3.6		49.0 ± 3.8	48.1 ±3.6	
Age range	21–72	27–70		24–77	24–63	

### DNA sequencing

A two-step "boost/nest" polymerase chain reaction (PCR) strategy was used to sequence the 18 kb promoter regions at Polymorphic DNA Technologies, Inc (Alameda, CA). We first did a boost reaction for a larger PCR amplicon and then used this amplicon as a template for the nest reaction, followed by sequencing of the nest product. The conditions for the boost PCR reaction were identical to the nest with the following exceptions: 10 ng of genomic DNA was used for the boost, then 1 μL of boost product as template for the nest reaction. The two reactions used two different pairs of different primers. PCR cycle was: 94°C for 4 min, 25 cycles of 94°C for 20 sec, 55°C for 25 sec and 72°C for 1 min, followed by an extension of 72°C for 7 min. Double-stranded DNA sequencing was carried out by using the Applied Biosystems 3730/3730*xl* chemistry in a 384-well format.

### Genotyping

A multiplex PCR-restriction fragment length polymorphism (RFLP) method was used for VNTR genotyping. PCR followed standard protocols, with Fast PCR Master Mix (Fermentas, Glen Burnie, MD, U.S.A.) as described before[76]. For -14kb-VNTR, the PCR product was 913-bp, 733-bp, 652-bp, and 771-bp for 1-, 2-, 3-, and 4-repeat respectively. 5’VNTR was 426-bp, 486-bp, 546-bp, and 606-bp for 6-, 7-, 8-, and 9-repeat respectively. Int8VNTR was 291-bp and 321-bp for 5- and 6-repeat, and 3’VNTR was 441-bp, 481-bp and 521-bp for 9-, 10- and 11-repeat. These VNTR’s PCR products were subject to agarose electrophoresis directly. The allele sequences of -10kb-pA (polyadenine) and simple sequence length polymorphism (SSLP) were verified by TA cloning (Invitrogen, Carlsbad, CA, U.S.A.) and DNA sequencing of TA clones (five to six per subject for a 95% confidence on biallelic polymorphisms). All Chromas sequencing graphs were refereed manually by two researchers independently for double-verification of sequence accuracy. All primers used are listed in S1 Table.

### Genetic analysis

Linkage disequilibrium (LD), expressed as D’ and r^2^[77], was analyzed by using Haploview (http://www.broadinstitute.org/haploview/haploview) for biallelic polymorphisms and SHEsis (http://analysis.bio-x.cn/myAnalysis.php) for multiallelic polymorphism[78, 79]. Haplotyper (http://www.people.fas.harvard.edu/~junliu/Haplo/click.html) and PHASE (http://www.stat.washington.edu/stephens/phase/download.2.0.2.html) softwares were used for haplotype inference[80]. Recombination fraction was estimated by LDhat (http://www.cecalc.ula.ve/BIOINFO/servicios/herr1/LDhat/readme.html).

To evaluate general chromosomal recombination, five populations of European ancestry in the 1000 Genomes Project (1KGP)[81, 82], including US Caucasians, Great Britain, Italy, Spain, and Finland, were combined for reliability to reveal recombination hotspots in *SLC6A3* by using the published FastEPRR protocol[83].

To localize genome wide association study (GWAS) markers in this gene, three SUDs GWAS datasets, all past their embargo periods, were downloaded from the dbGaP,[84] including Collaborative Study on the Genetics of Alcoholism [85] (COGA, phs000125.v1.p1), Study of Addiction: Genetics and Environment (SAGE, phs000092.v1.p1) and the Australian twin-family study of alcohol use disorder (OZALC, phs000181.v1.p1). Datasets were cleaned or quality-controlled extensively by using a published protocol,[86] followed by imputation as described before.[87] Basic manipulations of datasets used PLINK[88].

To estimate Tajima’s D statistic in 30 unrelated COGA subjects, we calculated nucleotide diversity θ as the number of segregating sites, S, divided by a_1_, where $a_{1}=\sum_{i=1}^{59}\frac{1}{i}=4.6632$, divided by the number of nucleotides sequenced. Heterogeneity π was estimated by $k=\sum_{j=1}^{S}2p_{j}(1-p_{j})$, divided by 1–(1/59) = 0.983051, divided by the number of nucleotides sequenced, where *p*_j_ was the observed frequency of the j^th^ diallelic polymorphism. Statistic D was calculated using the θ and π, according to Tajima[89]. Phylogenic analysis was carried out by ClustalX (http://www.bioinformatics.ubc.ca/resources/tools/index.php?name=clustalx), with neighbor joining method for clustering and phylogenetic tree was displayed by TreeView (http://taxonomy.zoology.gla.ac.uk/rod/treeview.html) for cladistic analysis[90, 91]. Validations used MEGA7[92] under the Kimura 2 parameter with Gamma Distributed as rates among sites model for nucleotide substitutions. The relatedness results were shown in a radiation graphic, rather than in traditional trees, for better visualization.

## Results

### Unique variation and ethnic differences

We sequenced the 18 kb promoter region including the 16 kb 5’ region, Exon 1, and the 2 kb Intron 1 and further genotyped the two VNTRs in Intron 8 and 3’UTR among 30 AAs and 30 EAs. A sample size of 30 allowed 80% confidence of detecting an allele with a frequency of 2.6% or 95% confidence of detecting an allele with a frequency of 4.9%. Based on more than 7,300 PCR reactions and sequencing of more than 2.4 mega-bases (Mb), the 60 subjects were found to carry 134 polymorphisms in the 18 kb 5’ promoter regions and 20.1% of them were novel (S2 Table). The 5’ promoter had two VNTRs, one novel at -14280 (-14kb-VNTR) and another at -11000 (5’VNTR/rs70957367); a novel -15.0kb-indel (insertion/deletion), one novel variable poly A at -10331 (-10kb-pA) and one novel SSLP at +1531 (use of the first base of Exon 1 as +1 and negative numbers for upstream of the promoter). All of these polymorphisms were all confirmed by TA cloning and DNA re-sequencing. The novel -14kb-VNTR had four alleles that were formed by multiple indels, di-nucleotide polymorphisms (DNPs) and single nucleotide polymorphisms (SNPs) (see S1 Fig *upper panel*). 5’VNTR had 7–9 repeats of imperfect 60 bp, whose primary sequence was reported previously[76]. -10kb-pA had 9–11 As. SSLP in Intron 1 had nine different length polymorphisms (S1 Fig *lower panel*). Int8VNTR had two alleles, 5 and 6 repeats of 30 bp and 3’VNTR had 9–11 repeats of 40 bp as previously discovered[8, 60, 93, 94].

**Fig 1:**
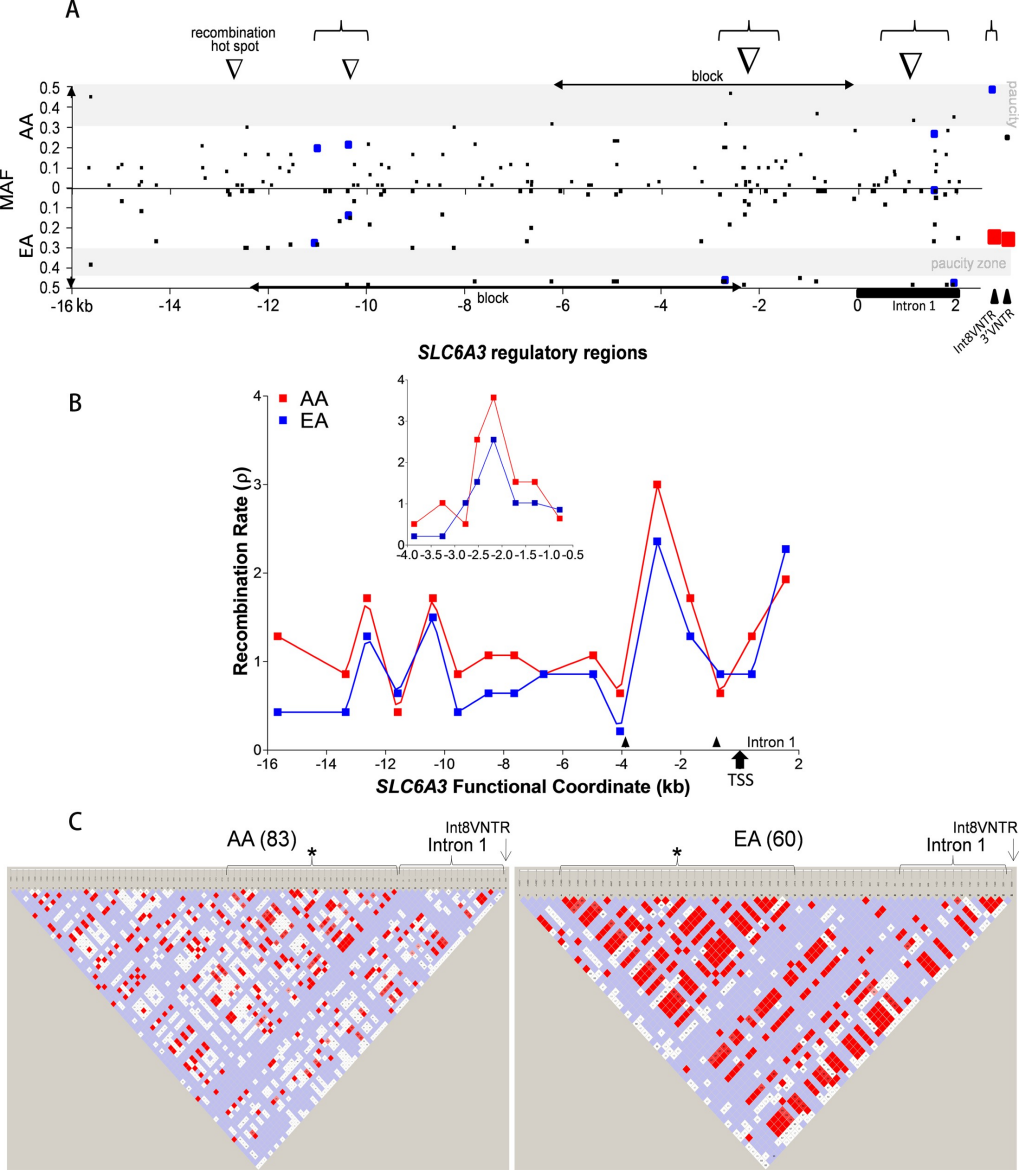
*SLC6A3* regulatory region polymorphisms. (A) Asymmetric distribution of common polymorphisms between the AA (*upper panel*) and the EA (*lower panel*) cohorts. Each polymorphism is indicated by small black square. Gray areas indicate paucity of polymorphisms for indicated MAF range. Black horizontal bar, location of Intron 1; black triangles, Int8VNTR and 3’VNTR; horizontal double-arrow, block; inverted open triangle, recombination hotspot for both populations; blue square, genetic selection; large red squares, SUDs-associated polymorphisms. Upper brace, clustering of selections. (B) Distribution of recombination rate across the 18 kb *SLC6A3* promoter regions. Red, AA; blue, EA. *Insert*, a close-up for the region indicated by two arrow heads, by using a finer scale (see x axis). Arrow, transcription start site (TSS). (C) Haploview-based linkage disequilibrium (LD) in *SLC6A3* regulatory regions (18 kb and Int8VNTR). *Left*, AA; *right*, EA. *, block; arrow, location of Int8VNTR. 3’VNTR was not included due to multiple alleles. Parenthesis contains the number of polymorphisms used for the LD analysis. Color: red for stronger LD; white, little LD. Brackets, haplotype blocks defined by Haploview.

For the sample size of 30 subjects, the AA group had 108 promoter polymorphisms and the EA group, 79 polymorphisms (see Fig 1A for distribution). The polymorphism density was 6.1/kb in AA and 4.5/kb in EA. Between AA and EA, 53 of the 134 polymorphisms were shared; the former carried 55 additional unique polymorphisms and the later carried 26 additional EA-specific ones. +24G/T (rs45611137) was the only Exon 1 mutant (minor allele frequency (MAF): 0.0167), present in the AA, not in the EA cohort.

### Recombination rate (*ρ*)

The core promoter region at -2.3 kb and Intron 1 at +1.6 kb both displayed higher recombination rates than upstream promoter regions (Fig 1B). Two other hot spots were at -10.4 kb and -12.6 kb. In the core promoter region, ***ρ*** was 3.57 in AA and 2.55 in EA, representing the hottest region for recombination in the *SLC6A3* promoter. This rate decreased sharply as the distance from the -2.3 kb spot increased towards either side (see *Insert* in Fig 1B). In this core promoter region, the average ***ρ*** value was 1.48 in the AA cohort, 1.4-fold higher of the ***ρ*** value in the EA cohort. The Intron 1 had another hotspot. The largest difference in recombination rate between the two cohorts was three-fold at -15.7 kb, the 5’ end of the promoter regions where ***ρ*** value was 1.29 in the AA and 0.43 in the EA cohort. The average ***ρ*** value was 1.26 in the AA, 1.3-fold higher of the ***ρ*** value in the EA cohort. Although we have not stratified the analysis by sex, the findings persistently pointed to four recombination hotspots in the *SLC6A3* promoter of the COGA cohorts.

### LD

The overall AA LD was low across the entire regulatory regions (average D’ = 0.8214 and square of the Pearson correlation coefficients r^2^ = 0.1283). There was a weak 6 kb block from -6234A/G (rs1354139) to -68T/A (rs2975226) (D’ = 0.8487 and r^2^ = 0.1621, see Fig 1C *left panel*). Intron 1 displayed relatively weak LD within the Intron (D’ = 0.8414 and r^2^ = 0.0964). In particular, the 5’ end of Intron 1 from +24G/T (rs45611137) to +579G/A (rs28382214) represented a major subregion of weak LD (D’ 0.8938 and r^2^ 0.0438). Int8VNTR displayed low LD (D’ 0.6126 and r^2^ 0.06524) with the upstream polymorphisms. Int8VNTR had perfect LD with 1787G/A (rs11564757) (D’ and r^2^ both = 1) and high LD with -15.0kb-indel, -10250C/T (rs72717506), -9701C/T (rs10063727), -4913A/G (rs10079467), -1675T/C (rs11564751) and -1479G/T (rs6413429) (D’ = 1 and r^2^ = 0.4).

The overall EA LD (D’ = 0.9119, r^2^ = 0.1876) was bit higher than the AA LD, consistent with the lower recombination rate in EA than in AA. There was a 10 kb block covering from -12499 to -2315, which was located towards the 5’ end, approximately 4 kb up compared to the location of the AA weak block (indicated by asterisks in Fig 1C *right panel*). The average LD within this block was D’ = 0.9458 and r^2^ = 0.2985. Polymorphisms at -5487, -6731, -3182, and -2600 displayed weak LD, D’ = 0.5333 and r^2^ = 0.06459 on average within this block. There is no LD between -10397G/A (rs6860992) and -4825T/C (rs188332761). Weak LD regions surrounded the transcription start site (TSS), covering the regions from -2296 to +1298 (D’ = 0.9507, r^2^ = 0.0835). Similar to what was observed in the AAs, the 5’ end of the EA Intron 1 also displayed weak LD. Different from the AAs was the 3’ end of Intron 1 that displayed strong LD within the region or with upstream regions. Again, Int8VNTR displayed weak LD (D’ = 0.6255, r^2^ = 0.08734) with upstream polymorphisms. However, Int8VNTR displayed strong LD with -15.0kb-indel, -10250C/T, -1675T/C (rs11564751) and +1787G/A (rs11564757) (D’ = 1 and r^2^ = 0.4).

When we stratified the 30 subjects by phenotypes, the control LD (D’ = 0.8774) was stronger than the case (D’ = 0.7992) in the AAs and differences in multifocal pairs were quite significant, by comparing the *upper panels* of S2 Fig. This phenotype-related difference in LD was also observed in EAs, especially for core promoter-Intron 1 *versus* the upstream regions (S2 Fig). Therefore, SUDs-related changes in LD might represent a major difference in *SLC6A3* genetics between AA and EA.

In addition to the biallelic polymorphisms, we also analyzed LD by using the five multiallelic polymorphisms, including -14kb-VNTR, 5’VNTR, -10kb-pA, SSLP and 3’VNTR. Consistently, the AA LD was weaker than the EA LD (S3 Fig *far right panels*). 3’VNTR displayed weak LD with upstream markers: r^2^ = 0.049–0.170 in the AAs and 0.115–0.200 in the EAs. Stratification with phenotypes showed that LD in these patients was stronger than in the controls for both populations, based on color intensity (S3 Fig *left panels*).

### Genetic selection of polymorphisms

Genetic selection may indicate functionality of polymorphisms. We utilized Tajima’s statistic D to evaluate these genetic processes. The average D values for the entire 18 kb promoter were slightly positive (0.48) for EA and slightly negative (-0.29) for AA. However, when we stratified the polymorphisms by variation types, significant D values were revealed. In the AA cohort, VNTRs and SSLP both had significantly positive D values. In particular, the Intron 1 SSLP (heterogeneity or h = 0.7556), 5’VNTR (h = 0.5915) and -10kb-pA (h = 0.6228) in the 5’ region had D values of 3.28, 2.67 and 2.48. Between two DNPs, the 5’ DNP had a negative D value but the Intron 1 DNP (DNPi) had a positive D (-1.09 and 1.47). The Intron 1 SSLP and DNP were the only types with positive D values in this region. Neither Int8VNTR nor 3’VNTR had significant D values (S4A Fig).

In the EA cohort, all types of variations (-10kb-pA, DNP, VNTR and SSLP) except SNPs had significantly positive D values (2.37, 2.32, 2.45, 4.57 *versus* -0.02857 for SNPs). This EA SSLP displayed the highest heterogeneity of 0.9647 among all of the polymorphisms. The positive DNP D was attributable to both DNPs in the 5’ region and in Intron 1. The positive VNTR D was attributable to 5’VNTR (h = 0.6395) in the 5’ region. Unlike the AA D values, SNPs and -15.0kb-indel were the only types that showed negative D values in the EA. The two VNTRs at the 3’ side of the gene showed positive but insignificant D values (S4B Fig). It was noticed that SSLP, 5’VNTR and -10kb-pA were all positively selected in both populations.

### Haplotypic relatedness

The AA and EA cohorts carried 108 and 79 polymorphisms plus Int8VNTR and 3’VNTR, and these polymorphisms constituted 57 different AA haplotypes (#1–57; #1-#29 from the patients and #30-#57 from the controls; #2, #34 and #40 each occurred twice in the controls) and 57 different EA haplotypes (#58-#114; #58-#87 from the patients and #88-#114 from the controls; #62 and #65 each occurred once in a control and once in a patients and #107 occurred twice in the two controls). That is, three haplotypes each occurred twice and 54 other haplotypes each occurred only once in each cohort of 30 subjects. None of the AA haplotypes were present in the EA and *vice versa*, suggesting that *SLC6A3* carries great diversity not only between different ethnicities but also within the same population.

**Fig 2:**
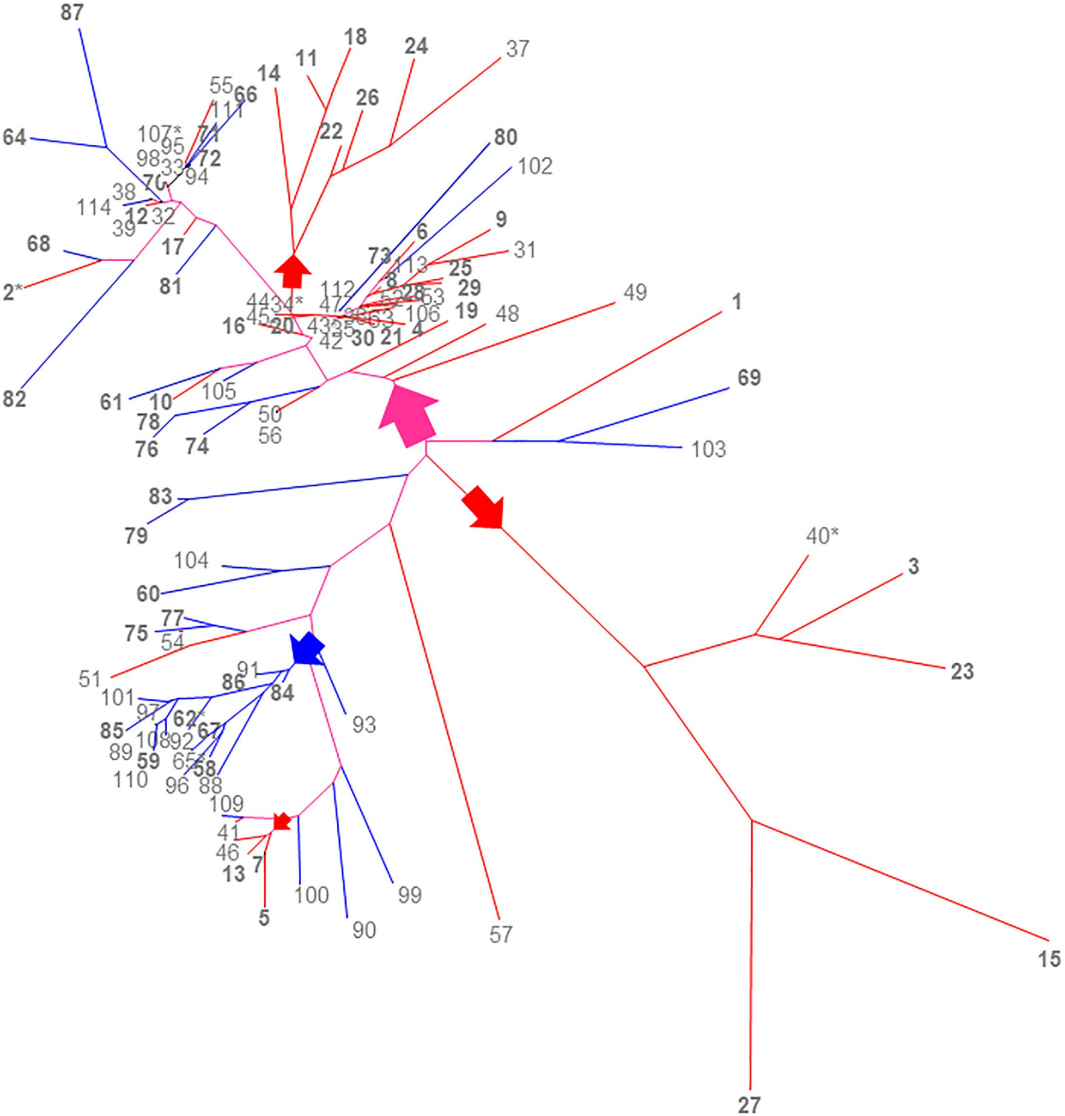
Unrooted neighbor-joining phylogenetic tree of AA (1–57 in red) and EA (58–114 in blue) regulatory haplotypes of *SLC6A3* (violet, shared branches). 1–29 and 58–87 (labeled in gray bold), carried by the patients and the rest by the controls. For top clade (at least five haplotypes), red arrows, AA clades; blue arrow, EA clade; violet arrow, clades with mixture of two ethnicities. *, carried by two subjects (the rest each carried by one subject). The proportion of sites where at least one unambiguous base was present in at least one haplotype for each descendent clade was either 99% or 100% for each internal node in the tree. The analyses utilized a total of 114 haplotypes.

Co-analyses of the AA and EA haplotypes for relatedness could help understand whether or not the *SLC6A3* haplotypes co-evolved independently between AA and EA during human history. Therefore, we generated a phylogenic tree containing all 114 haplotypes (Fig 2). It turned out that the AA and EA haplotypes were mixed up in terms of relatedness. Overall, the top half of the phylogenetic tree represented a major mosaic of the AA and EA haplotypes, including pairs of 10/61 and 2/68.

Haplotype analysis of three of the EA polymorphisms -14kb-VNTR_2/4_, Int8VNTR_5/6_ and 3’VNTR_9/10/11_ showed that 4-6-10 occurred eight times in the patients but did not occur in controls, with a nominal *p* value of 0.0046 by Fisher’s Exact Tests (odds ratio (OR) 23.0; 95% confidence interval (CI) 1.26–420.39). The consistencies in allele-specificity of association tendency and the suggestive haplotypic association warrant future investigation of these potential risk factors in large samples.

### Recombination hotspots in general populations of European ancestry

**Fig 3:**
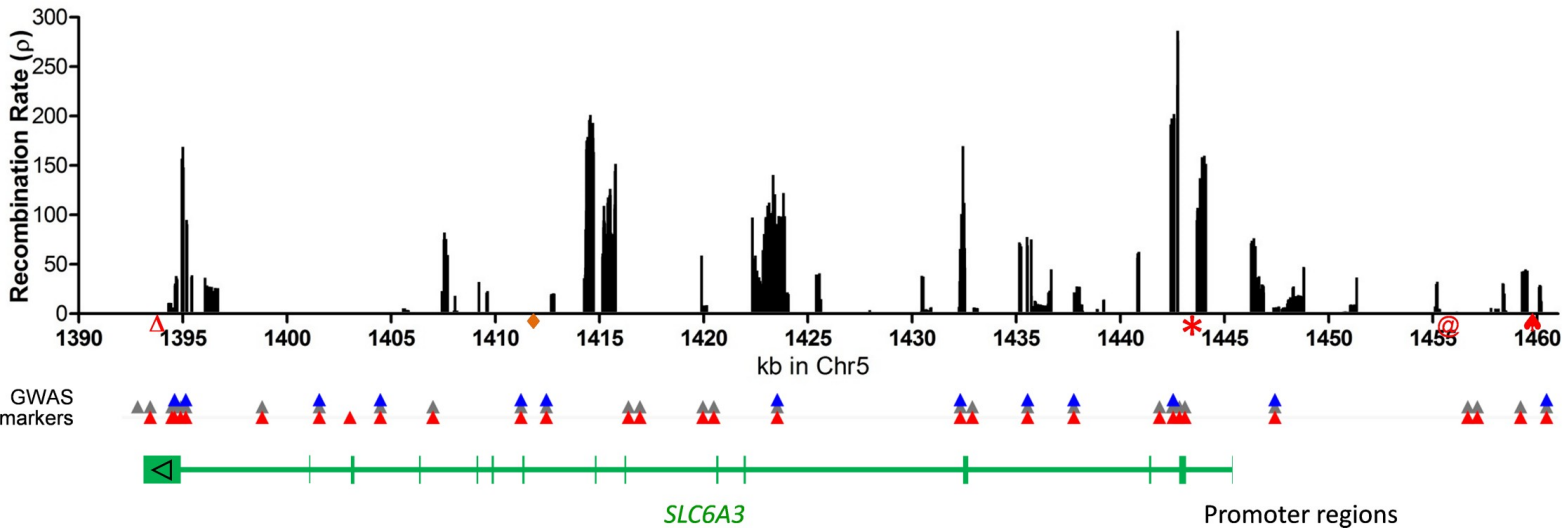
Caucasian recombination hotspots throughout *SLC6A3*. Recombination rate was obtained from combined five European ancestry populations (CEU, TSI, FIN, GBR and IBS for a total of 505 subjects) whose genotype data were collected by the 1000 Genomes Project. Indicated are also two frequently used genotyping markers, 3’VNTR (**△**), Intron 8 VNTR (◆), DNPi (*), 5’VNTR (@), -14kb-VNTR (♠) and effective GWAS markers (red ▲ for COGA, gray for SAGE and blue for the Australian twin-family study all from dbGaP; “effective” means surviving after the data quality control). Below the GWAS markers is *SLC6A3* gene structure in green (vertical bars are 15 exons in the opposite strand of the chromosome), localizing the polymorphisms and mostly intronic recombination hotspots in the gene. Six populations of African ancestry in the 1KGP (African Caribbean’s in Barbados (ACB), Americans in SW USA (ASW), Luhya in Webuye of Kenya (LWK), Esan (ESN) and Yoruba in Ibadan (YRI) of Nigeria, and Mende in Sierra Leone (MSL)) all carried the main hotspots at 1395, 1415, and 1443 (details not shown).

To confirm the recombination results from the COGA cohorts, we consulted with the 1KGP and analyzed the entire 70 kb chromosomal region of *SLC6A3*. We combined five European ethnicities, including EA (99 persons), Italy (108 persons), Spain (107 persons), Finland (99 persons), Great Britain (92 persons), for a total of 505 persons. Several hotspots were revealed but the most significant one was in Intron 2 and next to Exon 2. The ones in the core promoter and in Intron 1, revealed by the COGA subjects, were confirmed. Others were located in Introns 4, 7, 8, 11 and 14. None of them were localized to any coding regions. The distal promoter regions were relatively quiet, with six minor ones (Fig 3 *upper part*).

## Discussion

It is important to uncover novel, potentially functional polymorphisms and distinct haplotypes, and recombination hotspots because *SLC6A3* activity can be haplotype-dependent partly due to *cis*-antagonism between 5’ and 3’ sides of *SLC6A3*[95]. The most significant findings from this deep-sequencing study included discovery of novel and selected polymorphisms and the presence of recombination hotspots throughout *SLC6A3*. Although association analysis of SUDs and *SLC6A3* haplotypes was not a primary purpose here, five implications are highlighted as follows.

### Great diversity

The levels of variation including novel polymorphisms in the regulatory regions have never been expected. In either of the two cohorts, 60 chromosomes were represented by 57 regulatory haplotypes. The haplotypic diversity was attributable to both high density of polymorphisms and several recombination hotspots. Because of the genetic diversity, the overall LD was generally low, based on the r^2^ values of < 0.2 across the regulatory regions and the previously observed balancing selection of promoter haplotypes disappeared here[96, 97]. Such information explains the inconsistent findings on associations between *SLC6A3* and ADHD from genetic studies that used markers located in “random” regions for different populations[98–101]. The rationale of choosing *SLC6A3* markers in most of the studies suffered from the lack of a mechanistic understanding of regulatory genetics in ethnic *SLC6A3* and because of that, the obtained results were more marker- and sample-dependent than phenotype-dependent. Our systemic observations suggest that use of markers that cover more regulatory regions could result in more consistent and positive associations, a notion that has been supported by two studies[42, 46].

### Convergence

Phylogenetic analysis indicated for the first time the convergence of the *SLC6A3* regulatory regions between the AAs and the EAs, which means that DNA sequence variation occurred in this promoter independently in the two ethnicities. Despite one main EA clade and three localized minor AA clades, the majority of the haplotypes between two populations were mixed by being localized to many subclades of various sizes. Given the observations that two thirds of the EA polymorphisms were shared by the AA and that the four 5’ recombination hotspots were shared between the two ethnicities, it is a reasonable assumption that *SLC6A3* evolves in a largely context-dependent manner. In addition, the mosaic patterns could also be attributable to history of gene flow between European male and African female ancestry [102]. Finally, this phylogenetic tree indicates a tendency of bidirectional diversion among the 114 18kb-haplotypes. By analyzing twenty six 1KGP populations together (thousands of haplotypes), a much stronger bidirectional diversion of the 18kb promoter was observed (without geographic correlation, data not shown), validating the COGA subjects-based finding and implying again a functional correlation between genetics of the *SLC6A3* promoter and related phenotypes[103].

### Ethnicity

As Fig 1C shows, the AAs had clearly overall lower LD than the EAs. Specifically, there were three lines of evidence showing genetic differences in *SLC6A3* between the AAs and the EAs. First, the AA gene had many more common polymorphisms (Fig 1A *upper panel*) than the EA gene (Fig 1A *lower panel*), noticing that most polymorphisms appeared clustered around the recombination hotspots (indicated as inverted triangles). Second, the AA gene lacked polymorphisms with MAF > 0.3 but the EA gene lacked polymorphisms with 0.3 < MAF < 0.45 (see gray areas in Fig 1A), suggesting that the AA mutations were less likely to be selected and that the commonest EA variants had been selected. The lack of mutation fixation might have been compensated by the higher mutation frequency in the AA *SLC6A3*. The third evidence was that a haplotype block covered the core promoter region in the AA gene but was larger and covered the center of the 5’ region in the EA gene. These genetic differences between the two populations are consistent with the fact that dopamine-related brain function has ethnic differences and suggest that association study needs to use different sets of markers for different populations. These potentially functional polymorphisms might have contributed to the observed genetic selection of particular polymorphisms (see upper braces in Fig 1A).

### Selection

Despite the haplotypic diversity, selections of polymorphisms were observed. In both populations, polymorphisms with MAF of 0.31–0.40 were de-selected. For the most common or ethnicity-specific polymorphisms (MAF of > 0.40), de-selection was the most significant in the AAs whereas selection was the most significant in the EAs, representing the largest difference between the two ethnicities. Tajima’s D statistic has been used in trying to identify functional polymorphisms[104, 105] and indicates here the selection of some polymorphisms in both cohorts. We now report indeed that Tajima’s D results are consistent with the functionality of DNPi because the EA DNPi was positively selected and this DNPi indeed mediates a long non-coding RNA (lncRNA) regulation of the *SLC6A3* promoter[75]. Significantly, SSLP in Intron 1, 5’VNTR and -10kb-pA were all selected in both populations, consistent with the fact that *SLC6A3 cis*-acting elements such as the 5 kb super enhancer (5KSE) can be localized to distal 5’ regions[95] and that 5’VNTR was positively correlated with the mRNA levels in postmortem dopamine neurons[76]. These positive selections thus generate testable hypotheses for functionality and associations.

### Localized recombination

*SLC6A3* is located at 1.4 Mb, near a telomere of chr5, and harbors several hotspots, consistent with previous observations that regions near telomeres tend to have higher recombination rates than those near centromeres[106–109]. The identified recombination hotspots not only help explain negative findings on *SLC6A3* in previous GWAS but also guide selection of new association markers. Fig 3 *lower part* indicates genetic markers used by three previous GWAS, in reference to the distribution of the recombination hotspots. It is noticed that all of these markers are separated from the core promoter by recombination hotspots. The two widely used VNTRs, located in last exon and Intron 8, are each separated from the promoter regions (also as previously suggested [110]) by several recombination hotspots, suggesting that these markers are unable to capture information effectively on varying promoter activity[51]. As such, the previous association studies have not effectively used genetic markers for this gene yet. The recently identified functional DNPi (indicated by an asterisk)[75] is buried in two hotspots, suggesting that none of the previously used markers could capture the DNPi-related information. We have tried to use either the 1KGP or the COGA genotype as the templates to impute DNPi in the other GWAS datasets but failed completely. This is partly explained by the fact that DNPi has low LD with other markers and is located in a small, isolated haplotype block (S5 Fig). When sample sizes get large enough and using the 1KGP with strong LD as the template (S5 Fig), DNPi could be imputed but the results would not be reliable for cohorts of interest[111]. Therefore, this marker should be experimentally typed for accurate association information.

We acknowledge that the main limitations of this study include small sample sizes which aimed at common variants only, limited number of ethnicities and mainly the promoter regions. Including the patients might affect any ethnic comparisons and LD might cause overestimation of distances in the phylogenetic tree. The results, however, should provide guidance for rational selection of genetic markers for functional and association studies, as we have done before[51, 76], and for in-depth interrogations of the entire gene in more ethnicities.

## Conclusions

Extensive DNA sequence variations not only around the core promoter but also in other distal regulatory regions may work in concert or in haplotypes and influence dopamine-related individuality and diseases. Such genetic diversity of *SLC6A3* may help explain the elusiveness of previous association findings with the classical markers in the 3’ side. The findings also lay a foundation for a better understanding of the roles that the polymorphic *SLC6A3* plays in human brain.

## Supporting information

S1 Fig Details of multiple alleles of -14kb-VNTR (*upper panel*) and Intron 1 SSLP (*lower panel*).
(PDF)

S2 Fig Haploview-based linkage disequilibrium (LD) in *SLC6A3* regulatory regions by phenotypes of the two COGA cohorts. (PDF)

S3 Fig SHEsis-based LD by multi-allelic polymorphisms in the COGA cohorts. (PDF)

S4 Fig Genetic selection of polymorphisms by Tajima’s statistic D for AA (a) and EA (b).
(PDF)

S5 Fig Significant difference in 18kb *SLC6A3* promoter LD (D’) between 1KGP CEU (upper) and COGA sample (30 controls and 30 patients with SUDs). (PDF)

S1 Table PCR primers used. (XLSX)

S2 Table Common and unique alleles in COGA samples. (PDF)

## References

[1] Cerruti C, Walther DM, Kuhar MJ, Uhl GR. Dopamine transporter mRNA expression is intense in rat midbrain neurons and modest outside midbrain. Brain research Molecular brain research. 1993;18(1–2):181–6. Epub 1993/04/01. . 8479287 10.1016/0169-328x(93)90187-t

[2] Nirenberg MJ, Vaughan RA, Uhl GR, Kuhar MJ, Pickel VM. The dopamine transporter is localized to dendritic and axonal plasma membranes of nigrostriatal dopaminergic neurons. The Journal of neuroscience: the official journal of the Society for Neuroscience. 1996;16(2):436–47. Epub 1996/01/15. . 8551328 10.1523/JNEUROSCI.16-02-00436.1996 PMC6578661

[3] Nirenberg MJ, Chan J, Pohorille A, Vaughan RA, Uhl GR, Kuhar MJ, et al The dopamine transporter: comparative ultrastructure of dopaminergic axons in limbic and motor compartments of the nucleus accumbens. The Journal of neuroscience: the official journal of the Society for Neuroscience. 1997;17(18):6899–907. Epub 1997/09/15. . 9278525 10.1523/JNEUROSCI.17-18-06899.1997 PMC6573281

[4] Freed C, Revay R, Vaughan RA, Kriek E, Grant S, Uhl GR, et al Dopamine transporter immunoreactivity in rat brain. The Journal of comparative neurology. 1995;359(2):340–9. Epub 1995/08/21. 10.1002/cne.903590211 . 7499533

[5] Haber SN, Ryoo H, Cox C, Lu W. Subsets of midbrain dopaminergic neurons in monkeys are distinguished by different levels of mRNA for the dopamine transporter: comparison with the mRNA for the D2 receptor, tyrosine hydroxylase and calbindin immunoreactivity. The Journal of comparative neurology. 1995;362(3):400–10. Epub 1995/11/20. 10.1002/cne.903620308 . 8576447

[6] Hitri A, Casanova MF, Kleinman JE, Weinberger DR, Wyatt RJ. Age-related changes in [3H]GBR 12935 binding site density in the prefrontal cortex of controls and schizophrenics. Biological psychiatry. 1995;37(3):175–82. Epub 1995/02/01. 10.1016/0006-3223(94)00202-E . 7727626

[7] Myer NM, Boland JR, Faraone SV. Pharmacogenetics predictors of methylphenidate efficacy in childhood ADHD. Molecular psychiatry. 2017 Epub 2017/12/13. 10.1038/mp.2017.234 . 29230023 PMC7039663

[8] Vandenbergh DJ, Persico AM, Hawkins AL, Griffin CA, Li X, Jabs EW, et al Human dopamine transporter gene (DAT1) maps to chromosome 5p15.3 and displays a VNTR. Genomics. 1992;14(4):1104–6. Epub 1992/12/01. . 1478653 10.1016/s0888-7543(05)80138-7

[9] Vandenbergh DJ, Thompson MD, Cook EH, Bendahhou E, Nguyen T, Krasowski MD, et al Human dopamine transporter gene: coding region conservation among normal, Tourette's disorder, alcohol dependence and attention-deficit hyperactivity disorder populations. Molecular psychiatry. 2000;5(3):283–92. Epub 2000/07/13. . 10889531 10.1038/sj.mp.4000701

[10] Bi Y, Huang X, Niu W, Chen S, Wu X, Cao Y, et al No association between SLC6A2, SLC6A3, DRD2 polymorphisms and schizophrenia in the Han Chinese population. Psychiatry research. 2017;253:398–400. Epub 2017/04/30. 10.1016/j.psychres.2017.02.051 . 28454051

[11] Costa A, Riedel M, Muller U, Moller HJ, Ettinger U. Relationship between SLC6A3 genotype and striatal dopamine transporter availability: a meta-analysis of human single photon emission computed tomography studies. Synapse (New York, NY). 2011;65(10):998–1005. Epub 2011/03/16. 10.1002/syn.20927 . 21404331

[12] Gamma F, Faraone SV, Glatt SJ, Yeh YC, Tsuang MT. Meta-analysis shows schizophrenia is not associated with the 40-base-pair repeat polymorphism of the dopamine transporter gene. Schizophrenia research. 2005;73(1):55–8. Epub 2004/11/30. 10.1016/j.schres.2004.09.020 . 15567077

[13] Serretti A, Mandelli L. The genetics of bipolar disorder: genome 'hot regions,' genes, new potential candidates and future directions. Molecular psychiatry. 2008;13(8):742–71. Epub 2008/03/12. 10.1038/mp.2008.29 . 18332878

[14] Huang CC, Lu RB, Yen CH, Yeh YW, Chou HW, Kuo SC, et al Dopamine transporter gene may be associated with bipolar disorder and its personality traits. European archives of psychiatry and clinical neuroscience. 2015;265(4):281–90. Epub 2014/12/31. 10.1007/s00406-014-0570-0 . 25547317

[15] Ettinger U, Merten N, Kambeitz J. Meta-analysis of the association of the SLC6A3 3'-UTR VNTR with cognition. Neuroscience and biobehavioral reviews. 2016;60:72–81. Epub 2015/11/26. 10.1016/j.neubiorev.2015.09.021 . 26593110

[16] Onnink AM, Franke B, van Hulzen K, Zwiers MP, Mostert JC, Schene AH, et al Enlarged striatal volume in adults with ADHD carrying the 9–6 haplotype of the dopamine transporter gene DAT1. Journal of neural transmission (Vienna, Austria: 1996). 2016;123(8):905–15. Epub 2016/03/05. 10.1007/s00702-016-1521-x 26935821 PMC4969340

[17] Vaughan RA, Foster JD. Mechanisms of dopamine transporter regulation in normal and disease states. Trends in pharmacological sciences. 2013;34(9):489–96. Epub 2013/08/24. 10.1016/j.tips.2013.07.005 23968642 PMC3831354

[18] Thapar A, Holmes J, Poulton K, Harrington R. Genetic basis of attention deficit and hyperactivity. The British journal of psychiatry: the journal of mental science. 1999;174:105–11. Epub 1999/04/22. . 10211163 10.1192/bjp.174.2.105

[19] Gainetdinov RR, Caron MG. Genetics of childhood disorders: XXIV. ADHD, part 8: hyperdopaminergic mice as an animal model of ADHD. Journal of the American Academy of Child and Adolescent Psychiatry. 2001;40(3):380–2. Epub 2001/04/06. . 11288782 10.1097/00004583-200103000-00020

[20] Myer NM, Boland JR, Faraone SV. Pharmacogenetics predictors of methylphenidate efficacy in childhood ADHD. Molecular psychiatry. 2018;23(9):1–8. Epub 2017/12/13. 10.1038/mp.2017.234 . 29230023 PMC7039663

[21] Zhuang X, Oosting RS, Jones SR, Gainetdinov RR, Miller GW, Caron MG, et al Hyperactivity and impaired response habituation in hyperdopaminergic mice. Proceedings of the National Academy of Sciences of the United States of America. 2001;98(4):1982–7. Epub 2001/02/15. 10.1073/pnas.98.4.1982 11172062 PMC29368

[22] Bowton E, Saunders C, Erreger K, Sakrikar D, Matthies HJ, Sen N, et al Dysregulation of dopamine transporters via dopamine D2 autoreceptors triggers anomalous dopamine efflux associated with attention-deficit hyperactivity disorder. The Journal of neuroscience: the official journal of the Society for Neuroscience. 2010;30(17):6048–57. Epub 2010/04/30. 10.1523/jneurosci.5094-09.2010 20427663 PMC2881830

[23] Bonvicini C, Faraone SV, Scassellati C. Attention-deficit hyperactivity disorder in adults: A systematic review and meta-analysis of genetic, pharmacogenetic and biochemical studies. Molecular psychiatry. 2016;21(7):872–84. Epub 2016/05/25. 10.1038/mp.2016.74 27217152 PMC5414093

[24] Fonseca DJ, Mateus HE, Galvez JM, Forero DA, Talero-Gutierrez C, Velez-van-Meerbeke A. Lack of association of polymorphisms in six candidate genes in colombian adhd patients. Annals of neurosciences. 2015;22(4):217–21. Epub 2015/11/04. 10.5214/ans.0972.7531.220405 26526368 PMC4627201

[25] Soleimani R, Salehi Z, Soltanipour S, Hasandokht T, Jalali MM. SLC6A3 polymorphism and response to methylphenidate in children with ADHD: A systematic review and meta-analysis. American journal of medical genetics Part B, Neuropsychiatric genetics: the official publication of the International Society of Psychiatric Genetics. 2018;177(3):287–300. Epub 2017/11/25. 10.1002/ajmg.b.32613 . 29171685

[26] Moreau C, Meguig S, Corvol JC, Labreuche J, Vasseur F, Duhamel A, et al Polymorphism of the dopamine transporter type 1 gene modifies the treatment response in Parkinson's disease. Brain: a journal of neurology. 2015;138(Pt 5):1271–83. Epub 2015/03/26. 10.1093/brain/awv063 25805645 PMC5963414

[27] Guin D, Mishra MK, Talwar P, Rawat C, Kushwaha SS, Kukreti S, et al A systematic review and integrative approach to decode the common molecular link between levodopa response and Parkinson's disease. BMC medical genomics. 2017;10(1):56 Epub 2017/09/21. 10.1186/s12920-017-0291-0 28927418 PMC5606117

[28] Politi C, Ciccacci C, Novelli G, Borgiani P. Genetics and Treatment Response in Parkinson's Disease: An Update on Pharmacogenetic Studies. Neuromolecular medicine. 2018;20(1):1–17. Epub 2018/01/07. 10.1007/s12017-017-8473-7 . 29305687

[29] Robertson BD, Al Jaja AS, MacDonald AA, Hiebert NM, Tamjeedi R, Seergobin KN, et al SLC6A3 Polymorphism Predisposes to Dopamine Overdose in Parkinson's Disease. Frontiers in neurology. 2018;9:693 Epub 2018/09/07. 10.3389/fneur.2018.00693 30186226 PMC6110885

[30] Kaiser R, Hofer A, Grapengiesser A, Gasser T, Kupsch A, Roots I, et al L -dopa-induced adverse effects in PD and dopamine transporter gene polymorphism. Neurology. 2003;60(11):1750–5. Epub 2003/06/11. 10.1212/01.wnl.0000068009.32067.a1 . 12796525

[31] Kaplan N, Vituri A, Korczyn AD, Cohen OS, Inzelberg R, Yahalom G, et al Sequence variants in SLC6A3, DRD2, and BDNF genes and time to levodopa-induced dyskinesias in Parkinson's disease. Journal of molecular neuroscience: MN. 2014;53(2):183–8. Epub 2014/03/19. 10.1007/s12031-014-0276-9 . 24633632

[32] Contin M, Martinelli P, Mochi M, Albani F, Riva R, Scaglione C, et al Dopamine transporter gene polymorphism, spect imaging, and levodopa response in patients with Parkinson disease. Clinical neuropharmacology. 2004;27(3):111–5. Epub 2004/06/11. . 15190232 10.1097/00002826-200405000-00004

[33] Zhai D, Li S, Zhao Y, Lin Z. SLC6A3 is a risk factor for Parkinson's disease: a meta-analysis of sixteen years' studies. Neuroscience letters. 2014;564:99–104. Epub 2013/11/12. 10.1016/j.neulet.2013.10.060 24211691 PMC5352947

[34] Ahmed H, Abushouk AI, Gabr M, Negida A, Abdel-Daim MM. Parkinson's disease and pesticides: A meta-analysis of disease connection and genetic alterations. Biomedicine & pharmacotherapy = Biomedecine & pharmacotherapie. 2017;90:638–49. Epub 2017/04/17. 10.1016/j.biopha.2017.03.100 . 28412655

[35] Volkow ND, Wang GJ, Fischman MW, Foltin RW, Fowler JS, Abumrad NN, et al Relationship between subjective effects of cocaine and dopamine transporter occupancy. Nature. 1997;386(6627):827–30. Epub 1997/04/24. 10.1038/386827a0 . 9126740

[36] Dong C, Wong ML, Licinio J. Sequence variations of ABCB1, SLC6A2, SLC6A3, SLC6A4, CREB1, CRHR1 and NTRK2: association with major depression and antidepressant response in Mexican-Americans. Molecular psychiatry. 2009;14(12):1105–18. Epub 2009/10/22. 10.1038/mp.2009.92 19844206 PMC2834349

[37] Savelieva KV, Caudle WM, Findlay GS, Caron MG, Miller GW. Decreased ethanol preference and consumption in dopamine transporter female knock-out mice. Alcoholism, clinical and experimental research. 2002;26(6):758–64. Epub 2002/06/18. . 12068242

[38] Ashok AH, Marques TR, Jauhar S, Nour MM, Goodwin GM, Young AH, et al The dopamine hypothesis of bipolar affective disorder: the state of the art and implications for treatment. Molecular psychiatry. 2017;22(5):666–79. Epub 2017/03/16. 10.1038/mp.2017.16 28289283 PMC5401767

[39] Lohr KM, Masoud ST, Salahpour A, Miller GW. Membrane transporters as mediators of synaptic dopamine dynamics: implications for disease. The European journal of neuroscience. 2017;45(1):20–33. Epub 2016/08/16. 10.1111/ejn.13357 27520881 PMC5209277

[40] Gomez-Sanchez CI, Riveiro-Alvarez R, Soto-Insuga V, Rodrigo M, Tirado-Requero P, Mahillo-Fernandez I, et al Attention deficit hyperactivity disorder: genetic association study in a cohort of Spanish children. Behavioral and brain functions: BBF. 2016;12(1):2 Epub 2016/01/10. 10.1186/s12993-015-0084-6 26746237 PMC4706690

[41] de Azeredo LA, Rovaris DL, Mota NR, Polina ER, Marques FZ, Contini V, et al Further evidence for the association between a polymorphism in the promoter region of SLC6A3/DAT1 and ADHD: findings from a sample of adults. European archives of psychiatry and clinical neuroscience. 2014;264(5):401–8. Epub 2014/02/04. 10.1007/s00406-014-0486-8 . 24487615

[42] Doyle C, Brookes K, Simpson J, Park J, Scott S, Coghill DR, et al Replication of an association of a promoter polymorphism of the dopamine transporter gene and Attention Deficit Hyperactivity Disorder. Neuroscience letters. 2009;462(2):179–81. Epub 2009/07/07. 10.1016/j.neulet.2009.06.084 . 19576958

[43] Xu X, Mill J, Sun B, Chen CK, Huang YS, Wu YY, et al Association study of promoter polymorphisms at the dopamine transporter gene in Attention Deficit Hyperactivity Disorder. BMC psychiatry. 2009;9:3 Epub 2009/02/07. 10.1186/1471-244X-9-3 19196467 PMC2644291

[44] Genro JP, Zeni C, Polanczyk GV, Roman T, Rohde LA, Hutz MH. A promoter polymorphism (-839 C > T) at the dopamine transporter gene is associated with attention deficit/hyperactivity disorder in Brazilian children. American journal of medical genetics Part B, Neuropsychiatric genetics: the official publication of the International Society of Psychiatric Genetics. 2007;144b(2):215–9. Epub 2006/10/18. 10.1002/ajmg.b.30428 . 17044101

[45] Genro JP, Polanczyk GV, Zeni C, Oliveira AS, Roman T, Rohde LA, et al A common haplotype at the dopamine transporter gene 5' region is associated with attention-deficit/hyperactivity disorder. American journal of medical genetics Part B, Neuropsychiatric genetics: the official publication of the International Society of Psychiatric Genetics. 2008;147b(8):1568–75. Epub 2008/09/20. 10.1002/ajmg.b.30863 . 18802919

[46] Brookes KJ, Xu X, Anney R, Franke B, Zhou K, Chen W, et al Association of ADHD with genetic variants in the 5'-region of the dopamine transporter gene: evidence for allelic heterogeneity. American journal of medical genetics Part B, Neuropsychiatric genetics: the official publication of the International Society of Psychiatric Genetics. 2008;147b(8):1519–23. Epub 2008/08/01. 10.1002/ajmg.b.30782 . 18668530

[47] Ohadi M, Shirazi E, Tehranidoosti M, Moghimi N, Keikhaee MR, Ehssani S, et al Attention-deficit/hyperactivity disorder (ADHD) association with the DAT1 core promoter -67 T allele. Brain research. 2006;1101(1):1–4. Epub 2006/06/20. 10.1016/j.brainres.2006.05.024 . 16782077

[48] Sacchetti P, Brownschidle LA, Granneman JG, Bannon MJ. Characterization of the 5'-flanking region of the human dopamine transporter gene. Brain research Molecular brain research. 1999;74(1–2):167–74. Epub 2000/01/21. . 10640687 10.1016/s0169-328x(99)00275-2

[49] Greenwood TA, Kelsoe JR. Promoter and intronic variants affect the transcriptional regulation of the human dopamine transporter gene. Genomics. 2003;82(5):511–20. Epub 2003/10/16. . 14559208 10.1016/s0888-7543(03)00142-3

[50] Kouzmenko AP, Pereira AM, Singh BS. Intronic sequences are involved in neural targeting of human dopamine transporter gene expression. Biochemical and biophysical research communications. 1997;240(3):807–11. Epub 1997/12/17. 10.1006/bbrc.1997.7754 . 9398650

[51] Zhao Y, Xiong N, Liu Y, Zhou Y, Li N, Qing H, et al Human dopamine transporter gene: differential regulation of 18-kb haplotypes. Pharmacogenomics. 2013;14(12):1481–94. Epub 2013/09/13. 10.2217/pgs.13.141 ; PubMed Central PMCID: PMCPmc3870138. 24024899 PMC3870138

[52] Hall FS, Sora I, Uhl GR. Sex-dependent modulation of ethanol consumption in vesicular monoamine transporter 2 (VMAT2) and dopamine transporter (DAT) knockout mice. Neuropsychopharmacology: official publication of the American College of Neuropsychopharmacology. 2003;28(4):620–8. Epub 2003/03/26. 10.1038/sj.npp.1300070 . 12655306

[53] Giros B, Jaber M, Jones SR, Wightman RM, Caron MG. Hyperlocomotion and indifference to cocaine and amphetamine in mice lacking the dopamine transporter. Nature. 1996;379(6566):606–12. Epub 1996/02/15. 10.1038/379606a0 . 8628395

[54] Perona MT, Waters S, Hall FS, Sora I, Lesch KP, Murphy DL, et al Animal models of depression in dopamine, serotonin, and norepinephrine transporter knockout mice: prominent effects of dopamine transporter deletions. Behavioural pharmacology. 2008;19(5–6):566–74. Epub 2008/08/12. 10.1097/FBP.0b013e32830cd80f 18690111 PMC2644662

[55] Ralph-Williams RJ, Paulus MP, Zhuang X, Hen R, Geyer MA. Valproate attenuates hyperactive and perseverative behaviors in mutant mice with a dysregulated dopamine system. Biological psychiatry. 2003;53(4):352–9. Epub 2003/02/15. . 12586455 10.1016/s0006-3223(02)01489-0

[56] Salahpour A, Medvedev IO, Beaulieu JM, Gainetdinov RR, Caron MG. Local knockdown of genes in the brain using small interfering RNA: a phenotypic comparison with knockout animals. Biological psychiatry. 2007;61(1):65–9. Epub 2006/05/23. 10.1016/j.biopsych.2006.03.020 . 16712807

[57] Fuke S, Suo S, Takahashi N, Koike H, Sasagawa N, Ishiura S. The VNTR polymorphism of the human dopamine transporter (DAT1) gene affects gene expression. The pharmacogenomics journal. 2001;1(2):152–6. Epub 2002/03/26. . 11911442 10.1038/sj.tpj.6500026

[58] Miller GM, De La Garza R 2nd, Novak MA, Madras BK. Single nucleotide polymorphisms distinguish multiple dopamine transporter alleles in primates: implications for association with attention deficit hyperactivity disorder and other neuropsychiatric disorders. Molecular psychiatry. 2001;6(1):50–8. Epub 2001/03/13. . 11244485 10.1038/sj.mp.4000809

[59] VanNess SH, Owens MJ, Kilts CD. The variable number of tandem repeats element in DAT1 regulates in vitro dopamine transporter density. BMC genetics. 2005;6:55 Epub 2005/11/29. 10.1186/1471-2156-6-55 16309561 PMC1325255

[60] Guindalini C, Howard M, Haddley K, Laranjeira R, Collier D, Ammar N, et al A dopamine transporter gene functional variant associated with cocaine abuse in a Brazilian sample. Proceedings of the National Academy of Sciences of the United States of America. 2006;103(12):4552–7. Epub 2006/03/16. 10.1073/pnas.0504789103 ; PubMed Central PMCID: PMCPmc1450209. 16537431 PMC1450209

[61] Hill M, Anney RJ, Gill M, Hawi Z. Functional analysis of intron 8 and 3' UTR variable number of tandem repeats of SLC6A3: differential activity of intron 8 variants. The pharmacogenomics journal. 2010;10(5):442–7. Epub 2009/12/24. 10.1038/tpj.2009.66 . 20029387

[62] Logue MW, Lancour D, Farrell J, Simkina I, Fallin MD, Lunetta KL, et al Targeted Sequencing of Alzheimer Disease Genes in African Americans Implicates Novel Risk Variants. Frontiers in neuroscience. 2018;12:592 Epub 2018/09/14. 10.3389/fnins.2018.00592 30210277 PMC6119822

[63] Li LX, Liu GL, Liu ZJ, Lu C, Wu ZY. Identification and functional characterization of two missense mutations in NDRG1 associated with Charcot-Marie-Tooth disease type 4D. Human mutation. 2017;38(11):1569–78. Epub 2017/08/05. 10.1002/humu.23309 . 28776325

[64] Wang L, Fan J, Francis JM, Georghiou G, Hergert S, Li S, et al Integrated single-cell genetic and transcriptional analysis suggests novel drivers of chronic lymphocytic leukemia. Genome research. 2017;27(8):1300–11. Epub 2017/07/07. 10.1101/gr.217331.116 28679620 PMC5538547

[65] Paulo P, Maia S, Pinto C, Pinto P, Monteiro A, Peixoto A, et al Targeted next generation sequencing identifies functionally deleterious germline mutations in novel genes in early-onset/familial prostate cancer. PLoS genetics. 2018;14(4):e1007355 Epub 2018/04/17. 10.1371/journal.pgen.1007355 29659569 PMC5919682

[66] Li J, Wang L, Guo H, Shi L, Zhang K, Tang M, et al Targeted sequencing and functional analysis reveal brain-size-related genes and their networks in autism spectrum disorders. Molecular psychiatry. 2017;22(9):1282–90. Epub 2017/08/24. 10.1038/mp.2017.140 . 28831199

[67] Alexander J, Potamianou H, Xing J, Deng L, Karagiannidis I, Tsetsos F, et al Targeted Re-Sequencing Approach of Candidate Genes Implicates Rare Potentially Functional Variants in Tourette Syndrome Etiology. Frontiers in neuroscience. 2016;10:428 Epub 2016/10/07. 10.3389/fnins.2016.00428 27708560 PMC5030307

[68] Karakaya M, Storbeck M, Strathmann EA, Delle Vedove A, Holker I, Altmueller J, et al Targeted sequencing with expanded gene profile enables high diagnostic yield in non-5q-spinal muscular atrophies. Human mutation. 2018;39(9):1284–98. Epub 2018/06/03. 10.1002/humu.23560 . 29858556

[69] Phuong MA, Mahardika GN. Targeted Sequencing of Venom Genes from Cone Snail Genomes Improves Understanding of Conotoxin Molecular Evolution. Molecular biology and evolution. 2018;35(5):1210–24. Epub 2018/03/08. 10.1093/molbev/msy034 29514313 PMC5913681

[70] Haas DW, Bradford Y, Verma A, Verma SS, Eron JJ, Gulick RM, et al Brain neurotransmitter transporter/receptor genomics and efavirenz central nervous system adverse events. Pharmacogenetics and genomics. 2018;28(7):179–87. Epub 2018/05/31. 10.1097/FPC.0000000000000341 29847509 PMC6010221

[71] Rincon-Perez I, Sanchez-Carmona AJ, Albert J, Hinojosa JA. The association of monoamine-related gene polymorphisms with behavioural correlates of response inhibition: A meta-analytic review. Neuroscience and biobehavioral reviews. 2018;84:49–62. Epub 2017/11/21. 10.1016/j.neubiorev.2017.11.009 . 29155230

[72] Schacht JP, Anton RF, McNamara PJ, Im Y, King AC. The dopamine transporter VNTR polymorphism moderates the relationship between acute response to alcohol and future alcohol use disorder symptoms. Addiction biology. 2018 Epub 2018/09/20. 10.1111/adb.12676 . 30230123

[73] Barrie ES, Pinsonneault JK, Sadee W, Hollway JA, Handen BL, Smith T, et al Testing genetic modifiers of behavior and response to atomoxetine in autism spectrum disorder with ADHD. Journal of developmental and physical disabilities. 2018;30(3):355–71. Epub 2018/09/11. 10.1007/s10882-018-9590-4 30197492 PMC6128165

[74] Schuckit MA, Hesselbrock V, Tipp J, Anthenelli R, Bucholz K, Radziminski S. A comparison of DSM-III-R, DSM-IV and ICD-10 substance use disorders diagnoses in 1922 men and women subjects in the COGA study. Collaborative Study on the Genetics of Alcoholism. Addiction (Abingdon, England). 1994;89(12):1629–38. Epub 1994/12/01. . 7866247 10.1111/j.1360-0443.1994.tb03764.x

[75] Liu K, Yu J, Zhao J, Zhou Y, Xiong N, Xu J, et al (AZI2)3'UTR Is a New SLC6A3 Downregulator Associated with an Epistatic Protection Against Substance Use Disorders. Molecular neurobiology. 2018;55(7):5611–22. Epub 2017/10/07. 10.1007/s12035-017-0781-2 28983843 PMC5886844

[76] Zhou Y, Michelhaugh SK, Schmidt CJ, Liu JS, Bannon MJ, Lin Z. Ventral midbrain correlation between genetic variation and expression of the dopamine transporter gene in cocaine-abusing versus non-abusing subjects. Addiction biology. 2014;19(1):122–31. Epub 2011/10/27. 10.1111/j.1369-1600.2011.00391.x ; PubMed Central PMCID: PMCPmc3270208. 22026501 PMC3270208

[77] VanLiere JM, Rosenberg NA. Mathematical properties of the r2 measure of linkage disequilibrium. Theoretical population biology. 2008;74(1):130–7. Epub 2008/06/24. 10.1016/j.tpb.2008.05.006 18572214 PMC2580747

[78] Barrett JC, Fry B, Maller J, Daly MJ. Haploview: analysis and visualization of LD and haplotype maps. Bioinformatics (Oxford, England). 2005;21(2):263–5. Epub 2004/08/07. 10.1093/bioinformatics/bth457 . 15297300

[79] Shi YY, He L. SHEsis, a powerful software platform for analyses of linkage disequilibrium, haplotype construction, and genetic association at polymorphism loci. Cell research. 2005;15(2):97–8. Epub 2005/03/03. 10.1038/sj.cr.7290272 . 15740637

[80] Niu T. Algorithms for inferring haplotypes. Genetic epidemiology. 2004;27(4):334–47. Epub 2004/09/16. 10.1002/gepi.20024 . 15368348

[81] Sudmant PH, Rausch T, Gardner EJ, Handsaker RE, Abyzov A, Huddleston J, et al An integrated map of structural variation in 2,504 human genomes. Nature. 2015;526(7571):75–81. Epub 2015/10/04. 10.1038/nature15394 26432246 PMC4617611

[82] Auton A, Brooks LD, Durbin RM, Garrison EP, Kang HM, Korbel JO, et al A global reference for human genetic variation. Nature. 2015;526(7571):68–74. Epub 2015/10/04. 10.1038/nature15393 26432245 PMC4750478

[83] Gao F, Ming C, Hu W, Li H. New Software for the Fast Estimation of Population Recombination Rates (FastEPRR) in the Genomic Era. G3 (Bethesda, Md). 2016;6(6):1563–71. Epub 2016/05/14. 10.1534/g3.116.028233 27172192 PMC4889653

[84] Mailman MD, Feolo M, Jin Y, Kimura M, Tryka K, Bagoutdinov R, et al The NCBI dbGaP database of genotypes and phenotypes. Nature genetics. 2007;39(10):1181–6. Epub 2007/09/28. 10.1038/ng1007-1181 17898773 PMC2031016

[85] Grucza RA, Bierut LJ. Co-occurring risk factors for alcohol dependence and habitual smoking: update on findings from the Collaborative Study on the Genetics of Alcoholism. Alcohol research & health: the journal of the National Institute on Alcohol Abuse and Alcoholism. 2006;29(3):172–8. Epub 2007/03/22. . 17373405 PMC6527048

[86] Anderson CA, Pettersson FH, Clarke GM, Cardon LR, Morris AP, Zondervan KT. Data quality control in genetic case-control association studies. Nature protocols. 2010;5(9):1564–73. Epub 2010/11/19. 10.1038/nprot.2010.116 ; PubMed Central PMCID: PMCPmc3025522. 21085122 PMC3025522

[87] Kennedy JL, Xiong N, Yu J, Zai CC, Pouget JG, Li J, et al Increased Nigral SLC6A3 Activity in Schizophrenia Patients: Findings From the Toronto-McLean Cohorts. Schizophrenia bulletin. 2016;42(3):772–81. Epub 2015/12/29. 10.1093/schbul/sbv191 26707863 PMC4838105

[88] Purcell S, Neale B, Todd-Brown K, Thomas L, Ferreira MA, Bender D, et al PLINK: a tool set for whole-genome association and population-based linkage analyses. American journal of human genetics. 2007;81(3):559–75. Epub 2007/08/19. 10.1086/519795 ; PubMed Central PMCID: PMCPmc1950838. 17701901 PMC1950838

[89] Tajima F. Statistical method for testing the neutral mutation hypothesis by DNA polymorphism. Genetics. 1989;123(3):585–95. Epub 1989/11/01. 2513255 10.1093/genetics/123.3.585 PMC1203831

[90] Thompson JD, Gibson TJ, Higgins DG. Multiple sequence alignment using ClustalW and ClustalX. Current protocols in bioinformatics. 2002;Chapter 2:Unit 2.3. Epub 2008/09/17. 10.1002/0471250953.bi0203s00 . 18792934

[91] Page RD. TreeView: an application to display phylogenetic trees on personal computers. Computer applications in the biosciences: CABIOS. 1996;12(4):357–8. Epub 1996/08/01. . 8902363 10.1093/bioinformatics/12.4.357

[92] Kumar S, Stecher G, Tamura K. MEGA7: Molecular Evolutionary Genetics Analysis Version 7.0 for Bigger Datasets. Molecular biology and evolution. 2016;33(7):1870–4. Epub 2016/03/24. 10.1093/molbev/msw054 . 27004904 PMC8210823

[93] Brookes KJ, Mill J, Guindalini C, Curran S, Xu X, Knight J, et al A common haplotype of the dopamine transporter gene associated with attention-deficit/hyperactivity disorder and interacting with maternal use of alcohol during pregnancy. Archives of general psychiatry. 2006;63(1):74–81. Epub 2006/01/04. 10.1001/archpsyc.63.1.74 . 16389200

[94] Byerley W, Hoff M, Holik J, Caron MG, Giros B. VNTR polymorphism for the human dopamine transporter gene (DAT1). Human molecular genetics. 1993;2(3):335 Epub 1993/03/01. 10.1093/hmg/2.3.335 . 8098980

[95] Zhao Y, Yu J, Zhao J, Chen X, Xiong N, Wang T, et al Intragenic Transcriptional cis-Antagonism Across SLC6A3. Molecular neurobiology. 2018 Epub 2018/09/28. 10.1007/s12035-018-1357-5 . 30259411 PMC6437023

[96] Drgon T, Lin Z, Wang GJ, Fowler J, Pablo J, Mash DC, et al Common human 5' dopamine transporter (SLC6A3) haplotypes yield varying expression levels in vivo. Cellular and molecular neurobiology. 2006;26(4–6):875–89. Epub 2006/05/20. 10.1007/s10571-006-9014-3 . 16710758 PMC11520624

[97] Kelada SN, Checkoway H, Kardia SL, Carlson CS, Costa-Mallen P, Eaton DL, et al 5' and 3' region variability in the dopamine transporter gene (SLC6A3), pesticide exposure and Parkinson's disease risk: a hypothesis-generating study. Human molecular genetics. 2006;15(20):3055–62. Epub 2006/09/12. 10.1093/hmg/ddl247 . 16963468

[98] Li D, Sham PC, Owen MJ, He L. Meta-analysis shows significant association between dopamine system genes and attention deficit hyperactivity disorder (ADHD). Human molecular genetics. 2006;15(14):2276–84. Epub 2006/06/16. 10.1093/hmg/ddl152 . 16774975

[99] Gizer IR, Ficks C, Waldman ID. Candidate gene studies of ADHD: a meta-analytic review. Human genetics. 2009;126(1):51–90. Epub 2009/06/10. 10.1007/s00439-009-0694-x . 19506906

[100] Franke B, Hoogman M, Arias Vasquez A, Heister JG, Savelkoul PJ, Naber M, et al Association of the dopamine transporter (SLC6A3/DAT1) gene 9–6 haplotype with adult ADHD. American journal of medical genetics Part B, Neuropsychiatric genetics: the official publication of the International Society of Psychiatric Genetics. 2008;147b(8):1576–9. Epub 2008/09/20. 10.1002/ajmg.b.30861 . 18802924

[101] Franke B, Vasquez AA, Johansson S, Hoogman M, Romanos J, Boreatti-Hummer A, et al Multicenter analysis of the SLC6A3/DAT1 VNTR haplotype in persistent ADHD suggests differential involvement of the gene in childhood and persistent ADHD. Neuropsychopharmacology: official publication of the American College of Neuropsychopharmacology. 2010;35(3):656–64. Epub 2009/11/06. 10.1038/npp.2009.170 ; PubMed Central PMCID: PMCPmc3055604. 19890261 PMC3055604

[102] Bryc K, Auton A, Nelson MR, Oksenberg JR, Hauser SL, Williams S, et al Genome-wide patterns of population structure and admixture in West Africans and African Americans. Proceedings of the National Academy of Sciences of the United States of America. 2010;107(2):786–91. Epub 2010/01/19. 10.1073/pnas.0909559107 20080753 PMC2818934

[103] Li J, Haddad R, Santos V, Bavan S, Luetje CW. Receptive range analysis of a mouse odorant receptor subfamily. Journal of neurochemistry. 2015;134(1):47–55. Epub 2015/03/17. 10.1111/jnc.13095 25772782 PMC4472571

[104] Carlson CS, Thomas DJ, Eberle MA, Swanson JE, Livingston RJ, Rieder MJ, et al Genomic regions exhibiting positive selection identified from dense genotype data. Genome research. 2005;15(11):1553–65. Epub 2005/10/28. 10.1101/gr.4326505 16251465 PMC1310643

[105] Siewert KM, Voight BF. Detecting Long-Term Balancing Selection Using Allele Frequency Correlation. Molecular biology and evolution. 2017;34(11):2996–3005. Epub 2017/10/06. 10.1093/molbev/msx209 28981714 PMC5850717

[106] Chen NWG, Thareau V, Ribeiro T, Magdelenat G, Ashfield T, Innes RW, et al Common Bean Subtelomeres Are Hot Spots of Recombination and Favor Resistance Gene Evolution. Frontiers in plant science. 2018;9:1185 Epub 2018/08/30. 10.3389/fpls.2018.01185 30154814 PMC6102362

[107] Myers S, Bottolo L, Freeman C, McVean G, Donnelly P. A fine-scale map of recombination rates and hotspots across the human genome. Science (New York, NY). 2005;310(5746):321–4. Epub 2005/10/15. 10.1126/science.1117196 . 16224025

[108] Mackiewicz D, de Oliveira PM, Moss de Oliveira S, Cebrat S. Distribution of recombination hotspots in the human genome—a comparison of computer simulations with real data. PloS one. 2013;8(6):e65272 Epub 2013/06/19. 10.1371/journal.pone.0065272 23776462 PMC3679075

[109] Halldorsson BV, Palsson G, Stefansson OA, Jonsson H, Hardarson MT, Eggertsson HP, et al Characterizing mutagenic effects of recombination through a sequence-level genetic map. Science (New York, NY). 2019;363(6425). Epub 2019/01/27. 10.1126/science.aau1043 . 30679340

[110] Shumay E, Chen J, Fowler JS, Volkow ND. Genotype and ancestry modulate brain's DAT availability in healthy humans. PloS one. 2011;6(8):e22754 Epub 2011/08/10. 10.1371/journal.pone.0022754 21826203 PMC3149615

[111] Clarke TK, Adams MJ, Davies G, Howard DM, Hall LS, Padmanabhan S, et al Genome-wide association study of alcohol consumption and genetic overlap with other health-related traits in UK Biobank (N = 112 117). Molecular psychiatry. 2017;22(10):1376–84. Epub 2017/09/25. 10.1038/mp.2017.153 28937693 PMC5622124

