# Supplementary material for: Presence of recombination hotspots throughout SLC6A3
Source: PLoS One. 2019 Jun 11;14(6):e0218129. doi: 10.1371/journal.pone.0218129 (PMC6559656; doi:10.1371/journal.pone.0218129)
Supplement: S1 Fig — Details of multiple alleles of -14kb-VNTR (upper panel) and Intron 1 SSLP (lower panel). (PDF) [file pone.0218129.s001.pdf]

-14kb-VNTR: Alignment of four alleles

```
VNTR14kb-1          acacacatgctcacatgcactcatgctgaggcttacacatgtacacatgctcacatgcac
VNTR14kb-2          acacacatgctcacatgcactcatgctgaggcttacacatgtacacatgctcacatgcac
VNTR14kb-4          acacacatgctcacatgcactcatgctgaggcttacacatgtgcacatgctcacatgcac
VNTR14kb-3          acacacatgctcacatgcactcatgctgaggcttacacatgtacacatgctcacatgcac
                    ***************************************** ******************

VNTR14kb-1          tcatgctgaggcttacacgtacacacatgctcacatgcactcatgctgaggcttacacat
VNTR14kb-2          tcatgctgaggcttacacgtacgcacatgctcacatgcacgcatgctgaggcttacacat
VNTR14kb-4          tcatgctgaggcttacacgtacacacatgctcacatgcactcatgctgaggcttacacat
VNTR14kb-3          tcatgctgaggcttacacgtacacacgtgctcacatgcacgcatgctgaggcttacacat
                    ********************** *** ************* *******************

VNTR14kb-1          gtacacatgctcacatacactcatgctgaggcttacacatgtacacacatgctcacatgc
VNTR14kb-2          gtacacatgctcacatacactcatgctgaggcttacacatgtac----------------
VNTR14kb-4          gtacacatgctcacatacactcatgctgaggcttacacatgtacacacatgctcacatgc
VNTR14kb-3          gtacacatgctcacatacactcatgctgaggcttacacatgtac----------------
                    ********************************************

VNTR14kb-1          actcatgctgaggcttacacatgtacacatgctcacatgcactcatgctgaggcttacac
VNTR14kb-2          ------------------------acacatgctcacatgcactcatgctgaggcttacac
VNTR14kb-4          actcatgctgaggcttacacatgtacacatgctcacatgcactcatgctgaggcttacac
VNTR14kb-3          ------------------------acgcatgctcacatgcactcatgctgaggcttacac
                                            ** *********************************

VNTR14kb-1          atgtacacacatgctaacatgcactcatgctgaggcttatgcatgtacacacatgctaac
VNTR14kb-2          atgtacacacatgctcacatgcactcatgctgaggcttacacatgtacaca---------
VNTR14kb-4          atgtacacacatgctaacatgcactcatgctgaggcttacacatgtacaca---------
VNTR14kb-3          atgtacacacatgctcacatgcactcatgctgaggcttacacatgtacaca---------
                    *************** ***********************  **********

VNTR14kb-1          atgcactcatgctgaggctcacatgcactcatgctgaggcttacacgtacacacgtgctc
VNTR14kb-2          -------------------------------------------------------tgctc
VNTR14kb-4          -------------------------------------------------------tgctc
VNTR14kb-3          -------------------------------------------------------tgctc
                                                                           *****

VNTR14kb-1          acatgcactcatgctgaggcttacacatgtacacacatgctcacatgcactcatgctgag
VNTR14kb-2          acatgcactcatgctgaggcttacacatgtacacacatgctaacatgcactcatgctgag
VNTR14kb-4          acatgcactcatgctgaggcttacacatgtacacacatgctaacatgcactcatgctgag
VNTR14kb-3          acatgcactcatgctgaggcttacacatgtacacacatgctaacatgcactcatgctgag
                    ***************************************** ******************

VNTR14kb-1          gcttacacatgtacacac--gctcacatgcactcatgctgaggctcacatgcactcatgc
VNTR14kb-2          gcttacgcatgtacacacatgctaacatgcactcatgctgaggctcacatgcactcatgc
VNTR14kb-4          gcttacgcatgtacacacatgctaacatgcactcatgctgaggctcacatgcactcatgc
VNTR14kb-3          gcttacgcatgtacacacatgctaacatgcactcatgctgaggctcacatgcactcatgc
                    ****** ***********  *** ************************************


VNTR14kb-1          tgaggcttacacatgtacacatgctcacatgcactcatgctgaggcttacatatgtacac
VNTR14kb-2          tgaggctttcacgtacacacgtgctcacatgcactcatgctgaggcttgcacatgtacac
```

```
VNTR14kb-4          tgaggcttacacgtacacacgtgctcacatgcactcatgctgaggcttacacatgtacac
VNTR14kb-3          tgaggcttacacgtacacacgtgctcacatgcactcatgctgaggcttacacatgtacac
                    ******** *** *  **** *************************** ** ********

VNTR14kb-1          acatgctcacatgcactcatgctgaggcttacacatgtacacatgctcacatgcactcat
VNTR14kb-2          acatgctcacatgcactcatgctgaggcttacacatgtacacacgctcacatgcactcat
VNTR14kb-4          acatgctcacatgcactcatgctgaggcttacacatgtacacacgcccacatgcactcat
VNTR14kb-3          acatgctcacatgcactcatgctgaggcttacacatgtacacacgctcacatgcactcat
                    ******************************************* ** *************

VNTR14kb-1          gctgaggcttacatgtacacacatgctcacatgcactcatgctgaggcttacacatgtac
VNTR14kb-2          gctgaggcttac------------------------------------------------
VNTR14kb-4          gctgaggcttac------------------------------------------------
VNTR14kb-3          gctgaggcttac------------------------------------------------
                    ************

VNTR14kb-1          acacatgctcacatgcactcatgctgaggcttacacatgtacacacgctcacatgcactc
VNTR14kb-2          --------------------------------acatgtacacacatgctcacatgcactc
VNTR14kb-4          --------------------------------acatgtacacacatgctcacatgcactc
VNTR14kb-3          --------------------------------acatgtacacacatgctcacatgcactc
                                                    ***  *  ***** **************

VNTR14kb-1          acgctgaggctcacatgcactcatgccgaggcttacacatgtacacacgctcacatgcac
VNTR14kb-2          atgctgaggctcacatgcactcatgctgaggtttacacatgtacacatgctcacatgcac
VNTR14kb-4          atgctgaggctcacatgcactcatgctgaggcttacacatgtacacatgctcacatgcac
VNTR14kb-3          atgctgaggctcacatgcactcatgctgaggcttacacatgtacacatgctcacatgcac
                    * ************************ **** *************** ************

VNTR14kb-1          tcatgctgaggc-tacatatgtacacacatgctcacatgcactcatgctgaggcttacac
VNTR14kb-2          tcatgctgaggcttacatatgtacacacatgctcacatgcactcatgctgaggcttacac
VNTR14kb-4          tcatgctgaggcttacatatgtacacacatgctcacatgcactcatgctgaggcttacac
VNTR14kb-3          tcatgctgaggcttacatatgtacacacatgctcacatgcactcatgccgaggctt----
                    ************ *********************************** *******

VNTR14kb-1          gtgtacacatgctcacatgcactcatgctgagnctnacatgtacacacatgctcacatgc
VNTR14kb-2          atgtacacacgctcacatgcactcatgctgaggcttacatgtacacacatgctcacatgc
VNTR14kb-4          atgtacacatgctcacatgcactcatgctgaggct-acatgtacacacatgctcacatgc
VNTR14kb-3          ------------------------------------------------------------


VNTR14kb-1          actcatgctgaggctt
VNTR14kb-2          actcatgccgaggctt
VNTR14kb-4          actcatgctgaggctt
VNTR14kb-3          ----------------
```

SSLP at +1531 has nine alleles in African and Caucasian Americans

1. $AAGA_4(CAA)_5A_5(CA)_4A_4$

2. $AAGA_4(CAA)_5A_4C_8A_5$

3. $A_7(CAA)_5A_5(CA)_4A_4$

4. $A_7(CAA)_5A_4C_8A_5$

5. $A_7(CAA)_4A_7(CA)_4A_4$

6. $A_7(CAA)_2A_3CA_9(CA)_4A_4$

7. $A_7(CAA)_4A_6C_8A_5$

8. $GA_7(CAA)_3A_7(CA)_6A_2$

9. $GA_4(CAA)_5A_5(CA)_4A_4$

S1 Fig. Details of multiple alleles of -14kb-VNTR (*upper panel*) and Intron 1 SSLP (*lower panel*).
