# Supplementary material for: Presence of recombination hotspots throughout SLC6A3
Source: PLoS One. 2019 Jun 11;14(6):e0218129. doi: 10.1371/journal.pone.0218129 (PMC6559656; doi:10.1371/journal.pone.0218129)
Supplement: S2 Fig — (PDF) [file pone.0218129.s002.pdf]

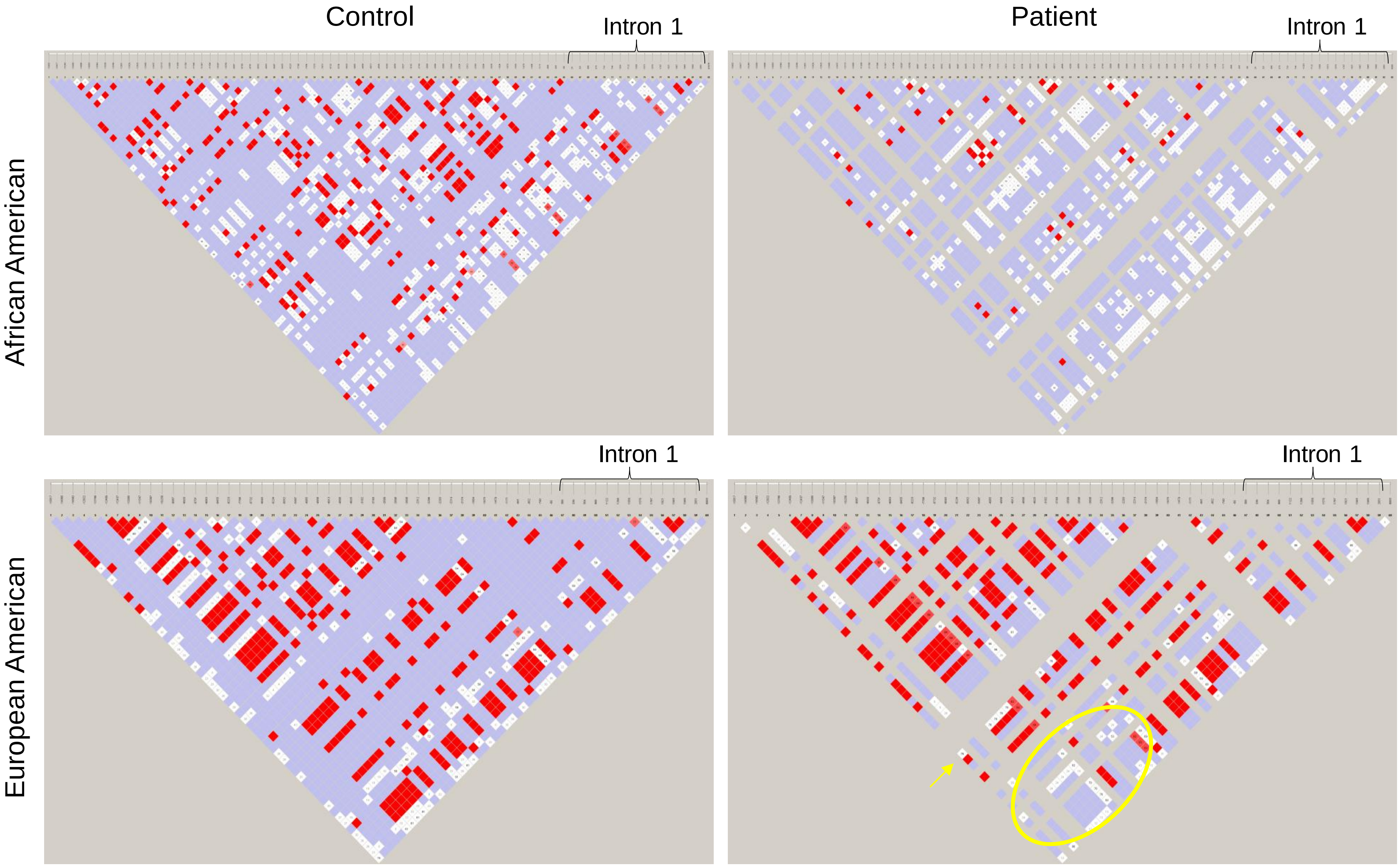

S2 Fig. Haploview-based linkage disequilibrium (LD) in *SLC6A3* regulatory regions by phenotypes of the two COGA cohorts. *Upper*, AA; *lower*, EA; *left*, controls and *right*, patient. Gray, absence of polymorphisms that were present in controls. Yellow, lower LD in patients than in controls.
