# Supplementary material for: Presence of recombination hotspots throughout SLC6A3
Source: PLoS One. 2019 Jun 11;14(6):e0218129. doi: 10.1371/journal.pone.0218129 (PMC6559656; doi:10.1371/journal.pone.0218129)
Supplement: S3 Fig — (PDF) [file pone.0218129.s003.pdf]

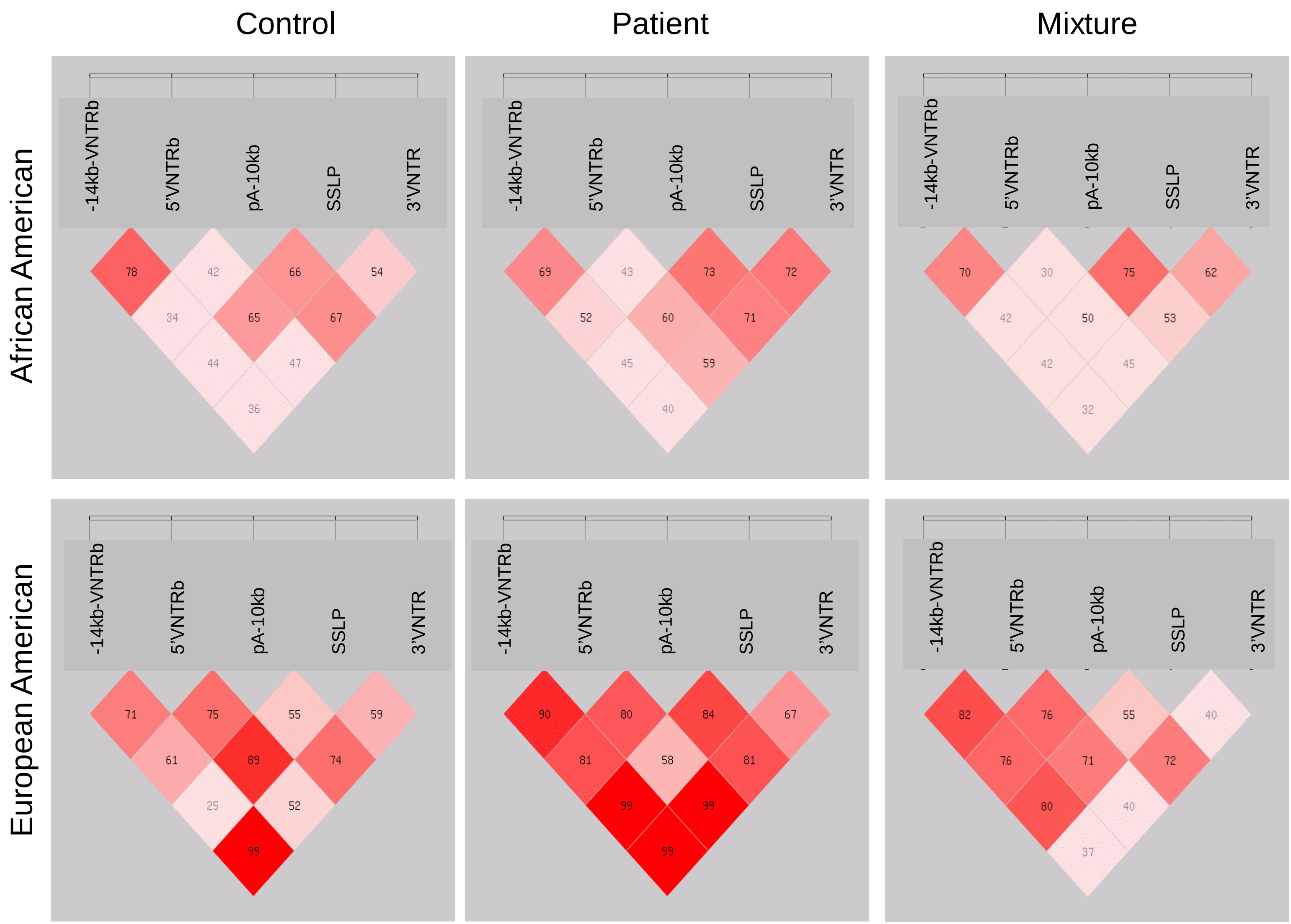

S3 Fig. SHEsis-based LD by multi-allelic polymorphisms in the COGA cohorts. In each panel, from left to right: -14kb-VNTR, 5'VNTR, pA-10kb, SSLP and 3'VNTR. Patient, with SUDs.
