# Supplementary material for: Presence of recombination hotspots throughout SLC6A3
Source: PLoS One. 2019 Jun 11;14(6):e0218129. doi: 10.1371/journal.pone.0218129 (PMC6559656; doi:10.1371/journal.pone.0218129)
Supplement: S4 Fig — Genetic selection of polymorphisms by Tajima’s statistic D for AA (a) and EA (b). (PDF) [file pone.0218129.s004.pdf]

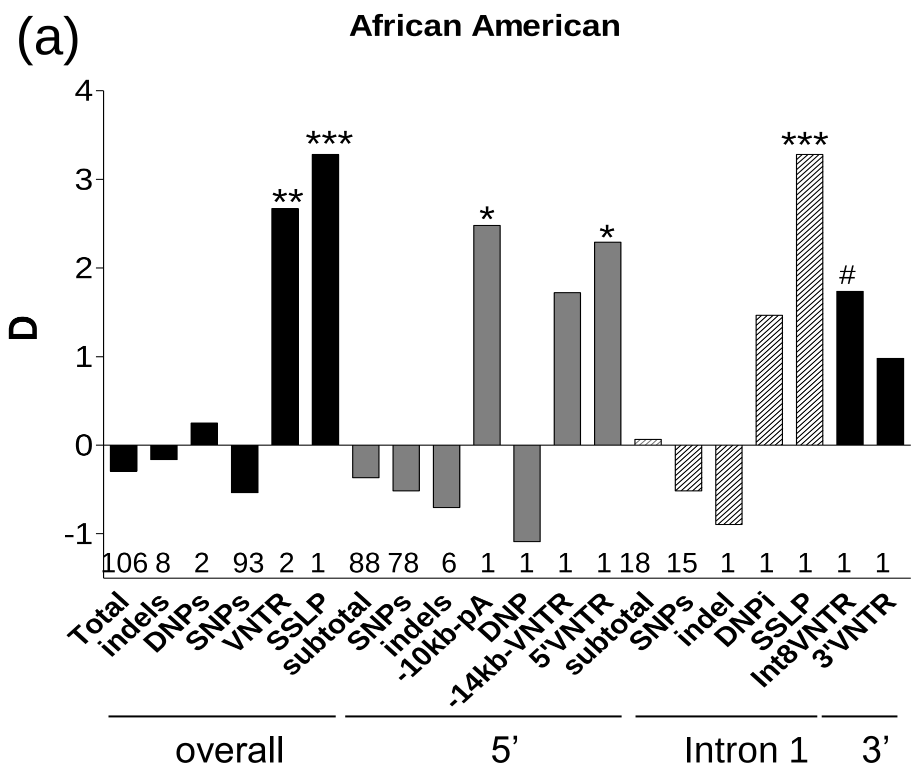

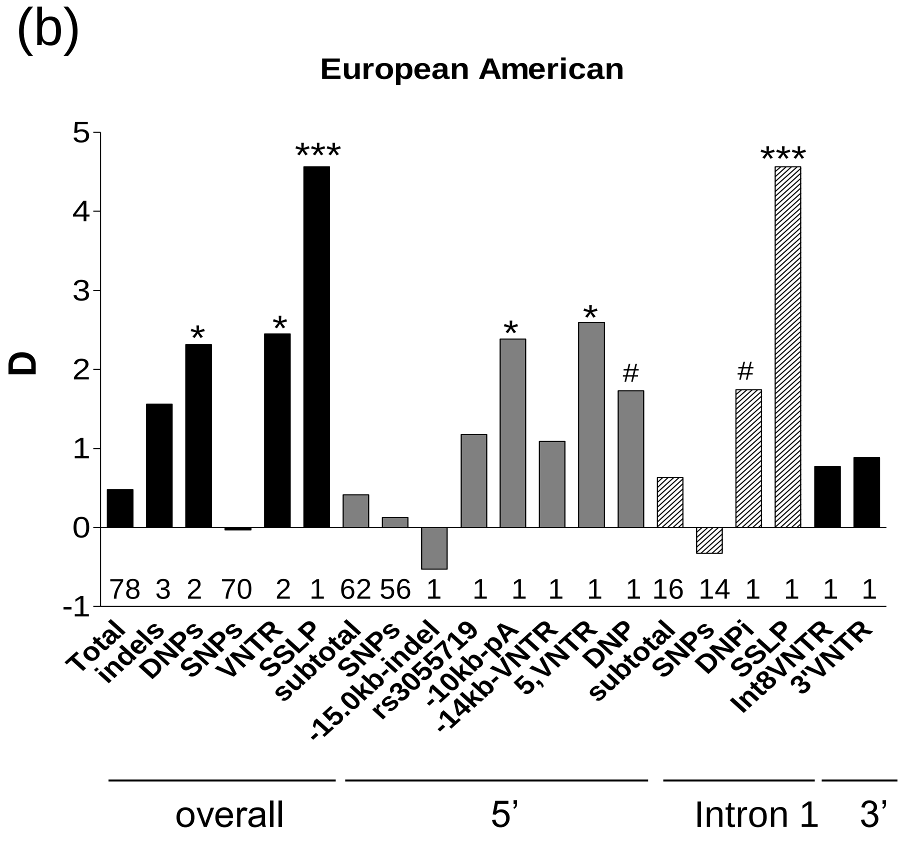

Fig S4. Genetic selection of polymorphisms by Tajima’s statistic D for AA (**a**) and EA (b). Polymorphisms are categorized by types for entire 18 kb regions (Overall, black bars where -10kb-pA is in indel), 5’ regions (5’, gray bars) and Intron 1 (hatched bars). Int8VNTR and 3’VNTR are listed on right side. #, $p < 0.1$; *, $p < 0.05$; **, $p < 0.01$; and ***, $p < 0.001$, based on β distribution.[38]
