# Supplementary material for: Presence of recombination hotspots throughout SLC6A3
Source: PLoS One. 2019 Jun 11;14(6):e0218129. doi: 10.1371/journal.pone.0218129 (PMC6559656; doi:10.1371/journal.pone.0218129)
Supplement: S5 Fig — (PDF) [file pone.0218129.s005.pdf]

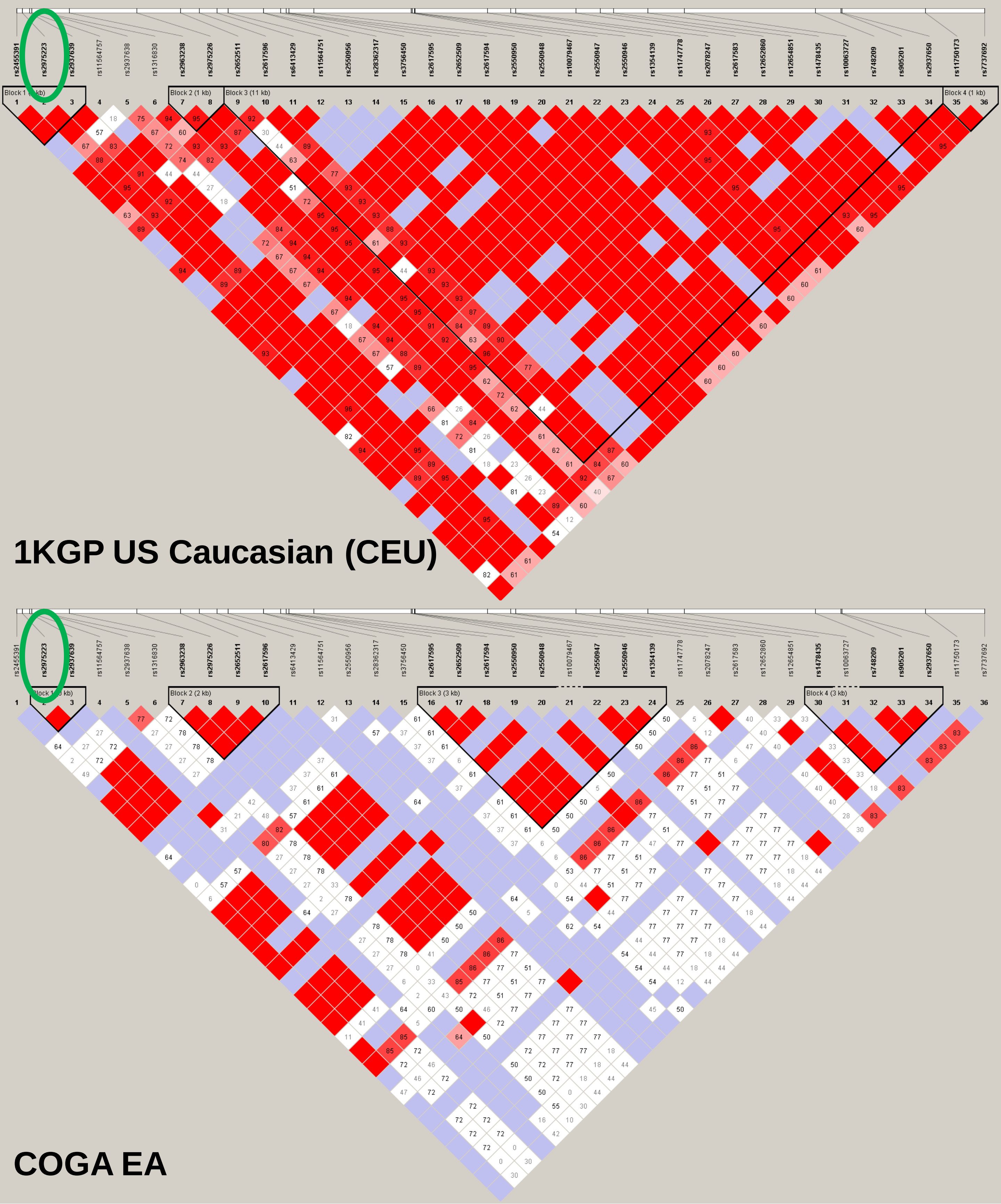

S5 Fig. Significant difference in 18 kb *SLC6A3* promoter LD (D’) between 1KGP CEU (upper) and COGA sample (30 controls and 30 patients with SUDs). Green circles rs2975223/DNPi.
