# Supplementary material for: Presence of recombination hotspots throughout SLC6A3
Source: PLoS One. 2019 Jun 11;14(6):e0218129. doi: 10.1371/journal.pone.0218129 (PMC6559656; doi:10.1371/journal.pone.0218129)
Supplement: S2 Table — (PDF) [file pone.0218129.s007.pdf]

S2 Table. Common and unique alleles in COGA samples.

chr5 position[a]	rs#	Functional position[b]	alleles major/minor	MAF AA	MAF EA
1443498	rs2455391	+2051	C/T	0.0333	0.25
1443532[c]	chr5:1443532	+2017	C/G	0	0.01666
1443603	rs2975223	+1946	A/G	0.35	0.51666
1443604	rs67175440	+1945	G/A	0.35	0.51666
1443702	rs12332463	+1847	G/A	0.0333	0
1443728	rs2937639	+1821	A/G	0.2833	0.51666
1443762	rs28382219	+1787	G/A	0.1666	0.0333
1443946	chr5:1443947	+1603	G/A	0	0.05
1443954	chr5:1443955	+1595	A/T	0	0.05
1443973	rs2937638	+1576	A/C	0.2666	0.81666
1443976	rs11564754	+1573	C/A	0.0833	0
1443990	rs1316830	+1559	A/G	0.18333	0.7333
1444018	SSLP	+1531	1/2,3,5,6,7,8,9	0.15/0.11666/0.35/0.2833	0.0333/0.18333/0/0/0/0.01666/0.01666
1444251	chr5:1444252	+1298	G/A	0	0.01666
1444427	rs2963238	+1122	A/C	0.333	0.51666
1444518	+1kb-indel	+1031	(GAGT)1/2	0.0333	0
1444581	rs28382215	+968	T/C	0	0.01666
1444805	rs116037986	+744	G/A	0.0666	0
1444817	rs2911493	+732	G/A	0.0833	0
1444970	rs28382214	+579	G/A	0.05	0
1444985	rs2735857	+564	G/T	0.0333	0.08333
1445141	rs370269420	+408	G/T	0.0166	0
1445210	chr5:1445211	+339	G/C	0	0.0166

1445228	chr5:1445229	+321	G/A	0.01666	0
1445240	chr5:1445241	+309	G/C	0	0.0166
1445525	rs45611137	+24	G/T	0.01666	0
1445617	rs2975226	-68	T/A	0.28333	0.055
1446210	rs113362701	-661	C/G	0	0.01666
1446224	rs2735852	-675	G/A	0.0333	0
1446289	rs536849094	-740	C/A	0	0.01666
1446351	rs115964423	-802	C/T	0	0.01666
1446390	rs2652511	-841	T/C	0.3666	0.5333
1446445	rs574461033	-896	C/G	0.05	0
1446720	rs2617596	-1171	G/C	0.1666	0.55
1446953	chr5:1446954	-1404	G/T	0.0166	0
1447028	rs6413429	-1479	G/T	0.1333	0.0333
1447104	rs112555354	-1555	C/T	0.1	0
1447224	rs11564751	-1675	T/C	0.1333	0.0666
1447383	rs2937637	-1834	C/A	0.1	0
1447413	rs540351375	-1864	C/T	0	0.0166
1447575	rs2937636	-2026	C/T	0.1	0
1447603	rs7733388	-2054	C/T	0.0166	0
1447723	rs116211357	-2174	G/A	0	0.0166
1447763	rs11564749	-2214	C/G	0.0333	0.08333
1447842	rs2550956	-2293	C/T	0.0333	0.1333
1447845	rs28362317	-2296	A/T	0.0166	0.0666
1447861	rs11564749	-2312	G/A	0.2	0.5166
1447873	rs2937635	-2324	C/T	0.1	0
1447898	rs2975225	-2349	G/C	0.1	0
1448029	rs145806429	-2480	G/A	0.0166	0
1448149	rs3756450	-2600	T/C	0.4666	0.18333

1448247	rs2617595	-2698	G/A	0.2	0.5333
1448248	rs2652509	-2699	T/C	0.31666	0.5333
1448289	rs2617594	-2740	T/C	0.2	0.5333
1448347	rs138035505	-2798	G/T	0	0.0333
1448731	rs2550950	-3182	G/A	0.0333	0.2666
1448876	rs2963236	-3327	C/T	0.1	0
1449607	rs111664342	-4058	C/T	0.0333	0
1449818	rs146316575	-4269	G/A	0.0166	0
1450374	rs188332761	-4825	T/C	0	0.01666
1450445	rs2550948	-4896	G/A	0.2333	0.5333
1450462	rs10079467	-4913	A/G	0.1166	0.0333
1450507	rs2550947	-4958	A/G	0.2333	0.5333
1450514	rs2550946	-4965	T/C	0.2333	0.5333
1450982	-5kb-2A	-5433	(A)11/10	0.0166	0
1451036	rs145859386	-5487	G/A	0.0166	0.0333
1451382	rs115288709	-5833	C/T	0.0333	0
1451601	chr5:1451131	-6052	A/C	0	0.01666
1451783	rs1354139	-6234	A/G	0.3166	0.5333
1452194	rs11747778	-6645	G/A	0.0333	0.2
1452199	rs116380738	-6650	T/C	0.0166	0.01666
1452280	rs114959205	-6731	T/C	0.0333	0
1452281	rs2078247	-6732	C/G	0.1166	0.2666
1452414	rs141180644	-6865	G/A	0	0.01666
1452621	rs60987235	-7072	G/A	0.11666	0
1452824	rs2975224	-7275	G/A	0.1	0
1453003	chr5:1453003	-7454	TC/-	0.0166	0
1453191	rs113127698	-7642	C/G	0.05	0
1453251	rs563628594	-7702	A/G	0.0166	0

1453345	rs2617583	-7796	G/T	0.2166	0.4666
1453772	rs12652860	-8223	G/T	0.3	0.3
1454004	rs12654851	-8455	C/A	0.1666	0.1333
1454060	rs142832833	-8511	C/T	0.0166	0
1454288	rs539605060	-8739	C/T	0.0166	0
1454536	rs9312868	-8987	C/T	0.0333	0
1454609	rs546701427	-9060	C/T	0	0.01666
1454612	rs1478435	-9063	G/A	0.1666	0.3
1455101	rs1478434	-9552	G/A	0.1	0
1455187	chr5:1455188	-9638	A/G	0	0.01666
1455237	rs575581666	-9688	G/A	0.0166	0
1455250	rs10063727	-9701	C/T	0.11666	0.0333
1455329	chr5:1455330	-9780	G/A	0.0166	0
1455482	rs34813657	-9933	C/T	0.0666	0.1833
1455516	rs4639276	-9967	G/A	0.2166	0.51666
1455728	rs141802731	-10179	C/T	0.0166	0
1455799	rs72717506	-10250	C/T	0.11666	0.0666
1455820	chr5:1455820	-10271	T/C	0.0166	0
1455880	-10kb-pA	-10331	9/10/11	0.2833/0.5/0.2166	0.15/0.5/0.35
1455929	chr5:1455929	-10380	A/G	0	0.0166
1455946	rs6860992	-10397	G/A	0.2166	0.5166
1456094	chr5:1456094	-10545	A/T	0	00166
1456289	chr5:1456289	-10740	A/G	0	0.01666
1456390	chr5:1456390	-10841	T/C	0	0.0166
1456549	5'VNTR/rs70957367	-11000	9/7,8	0.2/0.2166	0.2833/0.4666
1457053	rs2927667	-11504	C/T	0.11666	0
1457096	rs3055719	-11547	GAAAGAAA/-	0.1666	0.28333
1457133	rs56595782	-11584	T/C	0.08333	0

1457317	rs2937649	-11768	A/G	0.1	0
1457488	rs180911129	-11939	C/T	0.01666	0
1457554	rs748209	-12005	G/T	0.1666	0.3
1457659	rs111306318	-12110	G/T	0.0333	0
1457870	rs74469586	-12321	C/G	0	0.01666
1457916	rs577218594	-12367	A/T	0	0.01666
1457986	rs905201	-12437	A/G	0.3	0.3
1458018	rs2937650	-12469	C/T	0.1666	0.3
1458082	rs191008023	-12533	A/G	0.0166	0.01666
1458172	rs538162580	-12623	C/A	0.0166	0
1458173	rs549689763	-12624	T/A	0.0166	0
1458189	rs113757913	-12640	C/G	0.0166	0
1458335	rs140756212	-12786	C/T	0	0.0333
1458371	chr5:1458371	-12822	C/T	0	0.01666
1458400	rs2937651	-12851	C/T	0.0833	0
1458838	rs549654983	-13289	G/A	0.05	0
1458888	-13kb-indel	-13339	AC/-	0.10	0
1458898	rs146717703	-13349	G/A	0.20833	0
1459829	-14kb-VNTR	-14280	2/1,3,4	0.0333/0.01666/0.31667	0/0/0.2666
1460131	rs11750173	-14582	G/A	0.03333	0.11666
1460132	rs2937652	-14583	C/T	0.10	0
1460177	rs115571588	-14628	G/A	0.01666	0
1460529	-15.0kb-indel	-14980	(TTTG)1/2	0.11666	0.0666
1460608	-15.1kb-indel	-15059	GGGCAGGGTGGC)1/	0.10	0
1460793	rs188477182	-15244	C/T	0.01666	0
1461166	rs7737692	-15617	T/C	0.45	0.383
1461201	rs16878553	-15652	A/T	0.1	0

[a] GRCh37.p13.

[b] TSS = 1.

[c] Highlight: 27 new polymorphisms in chr5: 20.1%.
